# A Global Metabolomic and Lipidomic Landscape of Human Plasma Across the Lifespan

**DOI:** 10.1111/acel.70316

**Published:** 2025-12-06

**Authors:** Xinru Liu, Tingting Liang, Rui Zhao, Mingming Zhu, Beibei Huang, Xiaobi Huang, Fang Ni

**Affiliations:** ^1^ State Key Laboratory of Immune Response and Immunotherapy, Department of Hematology, The First Affiliated Hospital of USTC, Center for Advanced Interdisciplinary Science and Biomedicine of IHM, Institute of Blood and Cell Therapy and Anhui Provincial Key Laboratory of Blood Research and Applications, Division of Life Sciences and Medicine University of Science and Technology of China Hefei China; ^2^ Institute of Immunology, Division of Life Sciences and Medicine University of Science and Technology of China Hefei China; ^3^ Department of Pathology, The First Affiliated Hospital of USTC, Division of Life Sciences and Medicine University of Science and Technology of China Hefei China; ^4^ Department of Pediatric Nephrology Children's Hospital of Anhui Medical University (Anhui Provincial Children's Hospital) Hefei China

**Keywords:** aging, aging clock, lifespan, linear analysis, lipidomics, metabolomics, multi‐omics

## Abstract

Understanding metabolic changes across the human lifespan is essential for addressing age‐related health challenges. However, comprehensive metabolomic and lipidomic analyses, particularly in human plasma, remain underexplored. Herein, we performed untargeted metabolomics and lipidomics profiling of plasma collected from 136 individuals aged 0–84 years. This analysis reveals distinct metabolic signatures across life stages, with newborns displaying unique sphingosine (SPH) profiles, while aging was found to be characterized by elevated amino acid levels and lipid imbalances. Notably, we identified linear and nonlinear metabolic trajectories across the lifespan, highlighting critical transition points reflecting the key stages of metabolic reprogramming. By integrating these metabolic patterns, we developed an “aging clock” based on plasma metabolite profiling, thus providing a powerful tool to predict biological age. These findings offer new insights into the dynamic metabolic landscape of aging, paving the way for targeted interventions to improve healthspan and prevent age‐related diseases.

## Graphical abstract

Liu et al. provide a comprehensive analysis of dynamic plasma metabolomic and lipidomic changes in humans across the lifespan, identifying age‐specific metabolic trajectories and developing a metabolite‐based aging clock, offering insights into metabolic reprogramming and potential biomarkers for aging‐related health interventions.

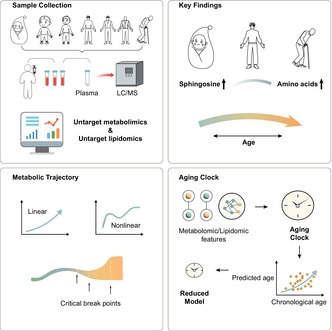

## 1 Introduction

Starting at birth and continuing throughout the process of aging, humans experience systematic changes in biological pathways and molecular profiles, accompanied by an increasing susceptibility to chronic diseases (Campisi et al. 2019; Furman et al. 2019; Guo et al. 2022; Hou et al. 2019). Although substantial progress has been made in understanding the mechanisms driving age‐related health risks, the complexity of these processes, which accumulate over a lifetime, remains a significant challenge.

Omics technologies, including proteomics (Lehallier et al. 2019; Oh et al. 2023; Tanaka et al. 2020; Tanaka et al. 2018), genomics (Aramillo Irizar et al. 2018; Debès et al. 2023; Stoeger et al. 2022; Tarkhov et al. 2024), microbiomics (Odendaal et al. 2024; Pang et al. 2023; Pu et al. 2024), and metabolomics (Hornburg et al. 2023; Wang et al. 2023; Wiley and Campisi 2021), have provided valuable insights into the biology of aging, highlighting key phenomena such as chronic inflammation and cellular senescence (Li, Li, et al. 2023; López‐Otín et al. 2023; Suryadevara et al. 2024) Advances in high‐throughput techniques have greatly enhanced the ability to detect molecular changes with greater precision. Among these, metabolomics stands out, as metabolites—the end products of cellular processes—offer direct insights into the physiological state of an organism (Johnson et al. 2016; Patti et al. 2012). This makes metabolomics a powerful tool for aging research. While prior studies have predominantly focused on specific metabolic pathways or age‐related changes within isolated age groups, a comprehensive understanding of how metabolism evolves across the human lifespan, particularly in terms of dynamic, systemic changes, remains largely unexplored.

Traditional metabolomic analyses have predominantly focused on hydrophilic compounds, while lipidomics, which examines hydrophobic lipids and related pathways, complements this by revealing the complexity of the lipidome (Wang et al. 2020; Wu et al. 2020). Plasma, as a biofluid, offers a unique snapshot of the body's metabolic state, reflecting global metabolic changes and localized alterations in tissue function (Cheng et al. 2015). Understanding how the metabolome of human plasma shifts throughout the lifespan is essential for addressing the increasing burden of age‐related diseases, such as cardiovascular disease, diabetes, and neurodegenerative disorders—conditions commonly associated with metabolic dysfunction. However, the full spectrum of metabolic alterations, as revealed by the untargeted metabolomic and lipidomic profiling of human plasma across the lifespan, is still poorly defined.

In this study, we present a comprehensive cross‐sectional atlas of metabolomic and lipidomic profiles spanning the human lifespan. By mapping the dynamic changes in plasma metabolites from birth to old age, we identified metabolic shifts regulating the physiological processes at different life stages. Specifically, our analyses uncovered distinctive metabolic signatures in newborns, as well as notable metabolic transitions from childhood to late adulthood. We further observed linear and nonlinear trajectories in plasma metabolites and lipids, highlighting key metabolic reprogramming events. Furthermore, by integrating omics data with clinical indicators, we were able to enhance the physiological interpretation of metabolic changes. Meanwhile, a metabolic‐lipidomic “aging clock” was established for the capability of predicting biological age. Overall, these findings provide new insights into the metabolic changes associated with aging and highlight potential avenues for interventions aimed at improving metabolic health and preventing age‐related diseases.

## 2 Results

### 2.1 Study Design and Metabolic Landscape of the Study Cohort

**FIGURE 1:**
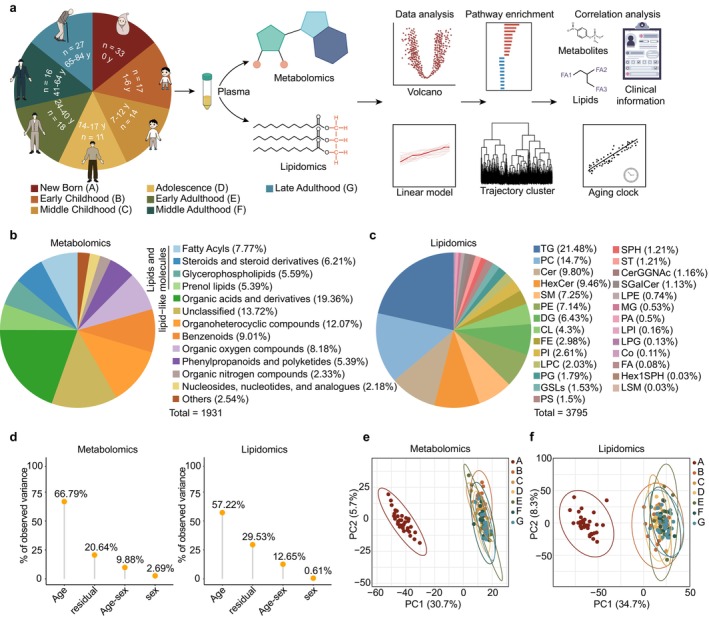
Schematic overview of the study design, and the metabolic landscape of the lifespan. (a) Flow chart of the study design, including sample collection and study overview. (b, c) Counts and classes of metabolites (b) and lipids (c) detected by LC–MS/MS in human plasma. Cer, Ceramide; CerGGNAc, Ceramide derivatives; CL, Cardiolipin; Co, Coenzyme; DG, diglyceride; FA, fatty acid; FE, fatty acid esters; GSLs, glycosphingolipids; Hex1SPH, hexosyl sphingosine; HexCer, Hexosyl ceramide; LPC, lysophosphatidylcholine; LPE, lysophosphatidylethanolamine; LPG, lysophosphatidylglycerol; LPI, lysophosphatidylinositol; LSM, lysosphingomyelin; MG, monoglyceride; PA, phosphatidic acid; PC, phosphatidylcholine; PE, phosphatidylethanolamine; PG, phosphatidylglycerol; PI, phosphatidylinositol; PS, phosphatidylserine; SGalCer, sulfatide; SM, sphingomyelin; SPH, Sphingosine; ST, Sterol; TG, triglyceride. (d) Visualization of the principal variance component analysis (PVCA), displaying the expression variance of the metabolites and lipids, as explained by residuals (technical and biological noise) and experimental factors (sex, age and age‐sex). “Sex” and “Age” represent the main effect of sex or age alone, respectively. “Age–sex” represents the proportion of variance explained by factors jointly associated with both age and sex. (e, f) Principal component analysis (PCA) score plots for metabolomics (e) and lipidomics (f) (*n* = 136 individuals) on all samples across the lifespan. Each sample is colored by group information.

To delve into the metabolic alterations in human plasma across the lifespan, we collected plasma samples from 136 volunteers aged 0–84 years. According to the stages of human development, we categorized the participants into seven groups (newborn, early childhood, middle childhood, adolescence, early adulthood, middle adulthood and late adulthood) (Figure 1a, Figure S1a, Table S1). The age distribution of the included participants is presented in Figure S1b. Using two extraction methods, we then performed untargeted metabolomic and lipidomic analyses, retaining 1931 metabolites across 13 classes and 3795 lipids across 27 classes after filtering (Figure 1a–c). All metabolites met the metabolomics standards initiative (MSI) Level 2 identification standards at minimum, including 469 Level 1 metabolites confirmed with authentic standards, while the remainder were identified as Level 2 based on MS/MS spectral matching. Overall, lipid‐related metabolites comprised the largest proportion in the metabolomic results, as their hydrophobic effects enable more accurate detection in untargeted lipidomics, further highlighting the necessity and scientific validity of our integrated multi‐omics approach. Lipids detected by lipidomics mainly included triglycerides (TGs), phospholipids such as phosphatidylcholine (PC) and phosphatidylethanolamine (PE), and sphingolipids such as ceramide (Cer), hexosyl ceramide (HexCer), and sphingomyelin (SM), comprehensively covering multiple pathway modules. To ensure data quality, we implemented rigorous quality control measures. Prior studies have noted the significant contribution of sex to human metabolomics (Austad and Fischer 2016; Bell et al. 2021; Li, Xiong, et al. 2023; Link and Reue 2017; Mauvais‐Jarvis 2024), prompting us to investigate its influence on our omics data. However, principal variance component analysis (PVCA) and principal component analysis (PCA) revealed only a minimal sex effect in metabolomics and lipidomics (2.69% and 0.61%, respectively) (Figure 1d, Figure S1c). Meanwhile, age was the factor with the greatest impact, that is, the largest proportion of global variance. Consequently, we suggest that while sex has a certain influence on metabolic changes across the lifespan, its impact is relatively small. To map the metabolic landscape at a broader level, we did not perform any sex‐stratified or age‐sex interaction in subsequent analyses. To visualize the holistic changes in metabolic data across the lifespan, PCA was applied on samples from all groups (Figure 1e,f). Strikingly, newborns were distinctly separated from all other stages, highlighting a marked difference in metabolic composition during this period. This suggests that the metabolic state undergoes substantial changes from birth through the developmental period. To further assess whether the strong effect of age was driven by the high distinction of the newborn group, we repeated the PVCA analysis after excluding newborn samples (Figure S1d). An increase in residual variance was observed, likely due to a reduction in age‐related variance when the highly distinct newborn group was removed; nevertheless, age remained the major biological contributor to variance, while sex accounted for the smallest proportion of the effect.

### 2.2 The Unique Metabolic Characteristics of Newborns

**FIGURE 2:**
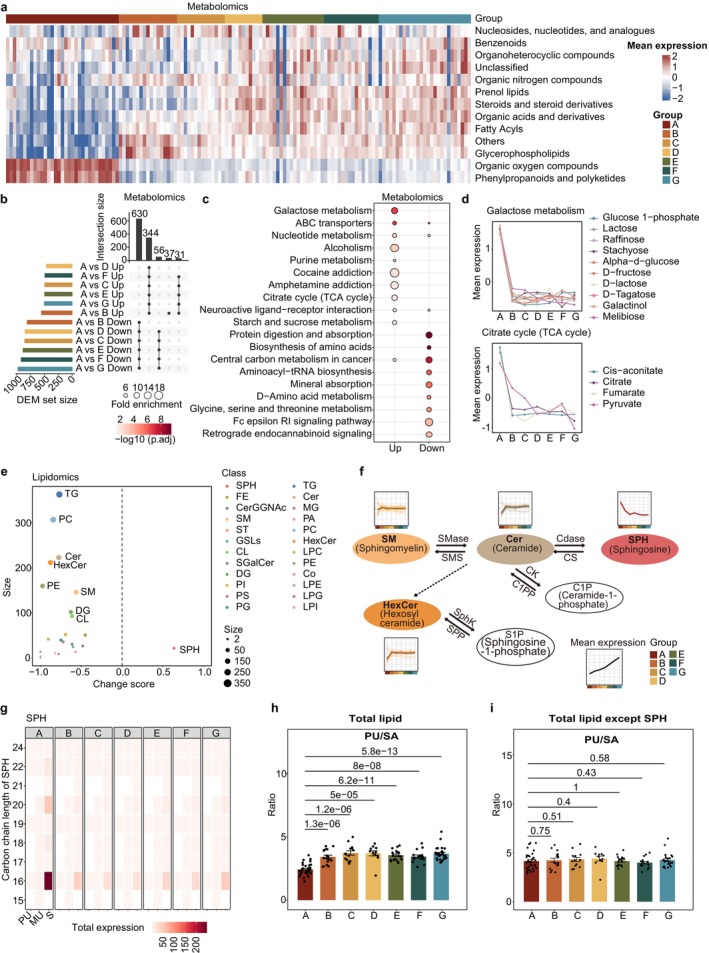
Significant perturbation in the newborn plasma metabolome. (a) Heatmap of the relative abundances of different metabolites in all samples. Color bars indicate the age groups. (b) UpSet plot showing the number of differentially expressed metabolites (DEMs) between newborns and other age groups. Statistical analyses were performed by two‐sided Student's *t*‐test and followed by Benjamini–Hochberg (BH) correction, and significantly changed metabolites were determined using BH‐corrected *p* value < 0.05. (c) Top‐10 Kyoto Encyclopedia of Genes and Genomes (KEGG) metabolic pathway enriched by metabolites up‐ and downregulated in newborns. The fisher's exact test (one‐sided) followed by BH‐corrected *p* value was applied. (d) Scaled mean expression of metabolites in the KEGG down pathway (Galactose metabolism and Citrate cycle). (e) New born‐related change score of different lipid classes. The dot size reflects the quantity of metabolites associated with age. (f) Metabolic pathway of SM and other related lipid subclasses with expression trajectory. (g) Heatmap showing the change of the carbon chain saturation (*x*‐axis) and carbon chain length (*y*‐axis) in SPH. The total expression represents the sum of normalized intensities of lipids containing the specified carbon chain length and number of double bonds. MU, monounsaturated (1 double bond); PU, polyunsaturated (≥ 2 double bonds); S, saturated (0 double bonds). (h) Bar graphs showing the ratios of concentrations of total polyunsaturated versus total saturated lipids in different age groups. (i) Bar graphs showing the ratios of concentrations of total polyunsaturated versus total saturated lipids excluding SPH in different age groups; each dot represents one biological replicate and presented as the mean ± SEM. Statistical significance was determined by two‐tailed unpaired *t*‐test.

Heatmaps were used to annotate the expression of major metabolite and lipid classes, clearly distinguishing newborns from other age groups, with opposing expression trends observed across various metabolite and lipid classes (Figure 2a, Figure S2a). Subsequently, we sought to identify which metabolites exhibited such distinct expression in newborns. Interestingly, differential expression metabolites (DEMs) were predominantly concentrated in the same categories, highlighting the reliable and consistent differential expression of these DEMs (Figure 2b, Figure S2b). We further applied median‐ratio normalization to confirm the consistency of the results. The newborn‐enriched metabolites identified were largely consistent with those obtained from total‐sum normalization (Figure S2c). Kyoto Encyclopedia of Genes and Genomes (KEGG) pathway enrichment analysis of these metabolites revealed significant alterations in multiple metabolic pathways (Figure 2c). Notably, galactose metabolism and the TCA cycle were upregulated in newborns, while metabolic pathways related to protein digestion and absorption, as well as the biosynthesis of amino acids were downregulated (Figure 2c,d, Figure S2d). This may be explained by the fact that glucose, which serves as the primary energy source for the fetus and is also the hydrolysis product of lactose, acts as a major substrate feeding into the TCA cycle and consequently supports the elevated energy demands of rapid postnatal growth and organ development. This further suggests that, newborns have a heightened metabolic rate to ensure adequate growth and organ development, as this life period is associated with rapid growth.

Moreover, among various lipids, sphingosine (SPH) is the only lipid class with elevated expression in newborns, while the expression of Cer and SM is lower (Figure 2e). This indicates that the conversion process from SMs to Cers and subsequently to SPHs may be more vigorous in cord blood (Figure 2f). Further, the conversion of Cer to HexCer was relatively low, collectively leading to the accumulation of higher levels of SPH. Understanding the variations in SPH content during developmental processes can further aid in identifying the onset of various diseases. Given that the key determinants of lipid function and characteristics are chain length and saturation (Zhang et al. 2024), a relevant analysis was performed. Intriguingly, we found that the saturation of plasma SPHs in newborns was significantly higher than in other age groups, particularly in long‐chain lipids with carbon chain lengths of 16, 20, and 22 (Figure 2g, Figure S2e). Consistently, the calculation of the total lipids revealed that the ratio of polyunsaturated to saturated lipids remained the lowest in newborns (Figure 2h). Interestingly, we found no difference in the saturation levels of all lipid classes following SPH removal, which to some extent reflects the pivotal role that SPH plays in newborns (Figure 2i). The degree of plasma lipid unsaturation gradually increases during human ontogeny from newborns, and has extraordinary significance in a variety of activities affecting vital biological functions, including cell membrane fluidity, signal transduction, metabolic regulation, and anti‐inflammatory effects. To explore the potential functional significance of elevated SPH levels in newborns, we conducted preliminary functional assays using cord blood mononuclear cells (CBMCs). Analysis revealed that treatment with SPH (10 μM, 24 h) markedly increased apoptosis across multiple immune cell subsets, including CD4^+^T, CD8^+^T, Natural Killer (NK), and B cells (Figure S3a,b). In addition, SPH treatment induced subset‐specific alterations in immune receptor expression (Figure S3c–g). SPH treatment upregulated activation markers (e.g., CD69) while concurrently reducing co‐stimulatory receptors (e.g., DNAM‐1, CD38) and enhancing inhibitory checkpoint molecules (e.g., PD‐1, TIGIT) in T cells, pointing to a state of activation‐induced stress and potential exhaustion. NK and B cells showed a decline in both activation and co‐stimulatory receptors, which likely reflects a transiently restrained immune state in newborns. These results indicate that SPH elevation can sensitize immune cells to apoptosis and modulate immune responsiveness, thereby potentially contributing to developmental immune remodeling in early life. Taken together, these data indicate that newborns exhibit highly unique metabolic profiles, characterized by a higher energy metabolism rate but concurrent with less mature lipid development, which was particularly reflected in the lower unsaturation levels of SPH. This suggests that a significant lipid remodeling process occurs postnatally.

### 2.3 Metabolic States Across Life Stages From Early Childhood to Late Adulthood

**FIGURE 3:**
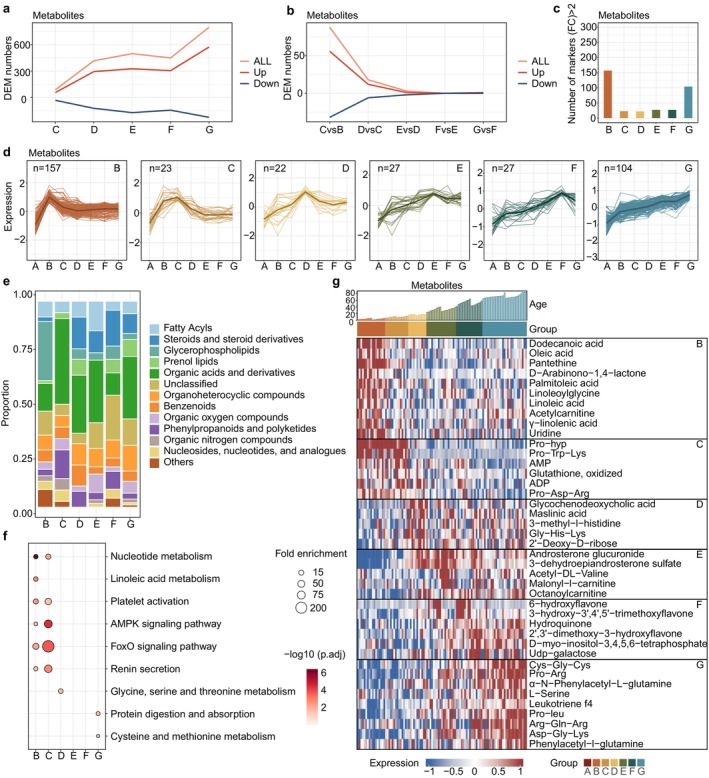
Metabolic characteristics of the different age groups. (a) Line plot showing the number of DEMs for pairwise analysis in age groups, referenced to early childhood (Group B). Each line is colored by the expression trends. (b) Line plot showing the number of DEMs for pairwise analysis in adjacent groups. Each line is colored by the expression trends. (c) Bar graph showing the number of upregulated metabolites for each group compared to all other groups. (d) Scaled mean expression trajectory of marker metabolites in age groups, with markers derived from (c). (e) Stacked bars illustrating the proportion of lipid classes of marker metabolites in age groups. (f) KEGG metabolic pathway enriched by marker metabolites in age groups. Fisher's exact test (one‐sided) followed by the BH‐corrected *p* value was used. (g) Scaled mean expression of selected marker metabolites in the different age groups. Color bars indicate the age groups; the top bar plot represents age.

Next, we sought to unravel the unique metabolic characteristics of each age group across the human lifespan. PCA revealed that, although the differences between groups were relatively small, samples from each stage gradually diverged from those of adjacent stages (Figure S4a), mirroring the progressive changes in metabolic composition that occur throughout the aging process. We were curious about the process and rate of these changes across the different stages. Therefore, we performed pairwise differential expression of metabolites to determine when and how changes in metabolic states occur across the human lifespan. Compared to early childhood (Group B), differences in metabolites and lipids progressively increased with age (Figure 3a, Figure S4b). Pairwise differential analysis of adjacent groups revealed that the metabolic changes were more pronounced during the rapid growth phases from early childhood to adolescence, while these changes gradually slowed down after middle age (Figure 3b, Figure S4c). Together, these data illustrate a fluctuating pattern of metabolic characteristics across the lifespan, with rapid changes occurring initially, followed by a gradual slowdown.

We further analyzed the most distinctive components representing each stage (Figure 3c–e, Figure S4d–f). Specifically, fatty acids (linoleic acid, oleic acid, palmitoleic acid, etc.) were significantly elevated in early childhood (Group B), while PC and PE were the predominant lipids in the lipidome. The increased levels of fatty acids may modulate the levels of PE and PC through various pathways, collectively reflecting the heightened activity of lipid metabolism during early childhood. In middle childhood (Group C), a stage characterized by significant physical growth and the gradual enhancement of physical strength and motor skills (Rogol et al. 2000), we consistently observed a marked increase in the levels of ADP and ATP, accompanied by an elevated presence of certain amino acids, particularly proline‐containing peptides.

A surge in the proportion of steroid and steroid derivatives among the elevated metabolites emerged in the transition into adolescence (Group D). Additionally, among the lipidome markers, TGs and diglyceride (DGs) were notably abundant. Steroids may regulate glycerolipid metabolism via hormonal regulation, thereby reflecting the impact of active sex hormone secretion during puberty on the complex interplay with various lipids, particularly glycerides. After early adulthood (Group E), metabolic changes were relatively small and closely resembled those in middle adulthood (Group F), indicating a more stable physiological condition. It is worth noting that Cer‐related lipids exhibit distinct characteristics during middle adulthood (Figure S4g). Prior studies have further reported that Cers are associated with neurodegenerative diseases (Trayssac et al. 2018), diabetes (Chaurasia et al. 2019), and cardiovascular diseases (Choi et al. 2021), indicating the importance of early monitoring of metabolic status at appropriate stages to prevent the onset of related diseases.

A plethora of highly expressed metabolites and lipids were identified in the final stage of the human lifespan (late adulthood, Group G) (Figure 3c, Figure S4d). Pathway enrichment and heatmap analysis revealed a notable increase in amino acid peptides (Figure 3f,g). This phenomenon reflects an active state of protein metabolism in late adulthood. Cysteine and glutamine, precursors of glutathione synthesis, perform crucial functions as key antioxidants in inflammation and immune responses (Gorrini et al. 2013). The increased plasma cysteine levels may be associated with a state of chronic inflammation, highlighting the intertwined roles of amino acid metabolism and anti‐inflammatory mechanisms in aging. In summary, these data delineate a metabolic landscape of uniquely upregulated representative metabolites across different life stages, thus elucidating the dynamic state transitions and physiological implications throughout the lifespan.

### 2.4 Linear Changes in the Metabolome and Lipidome Associated With Aging

**FIGURE 4:**
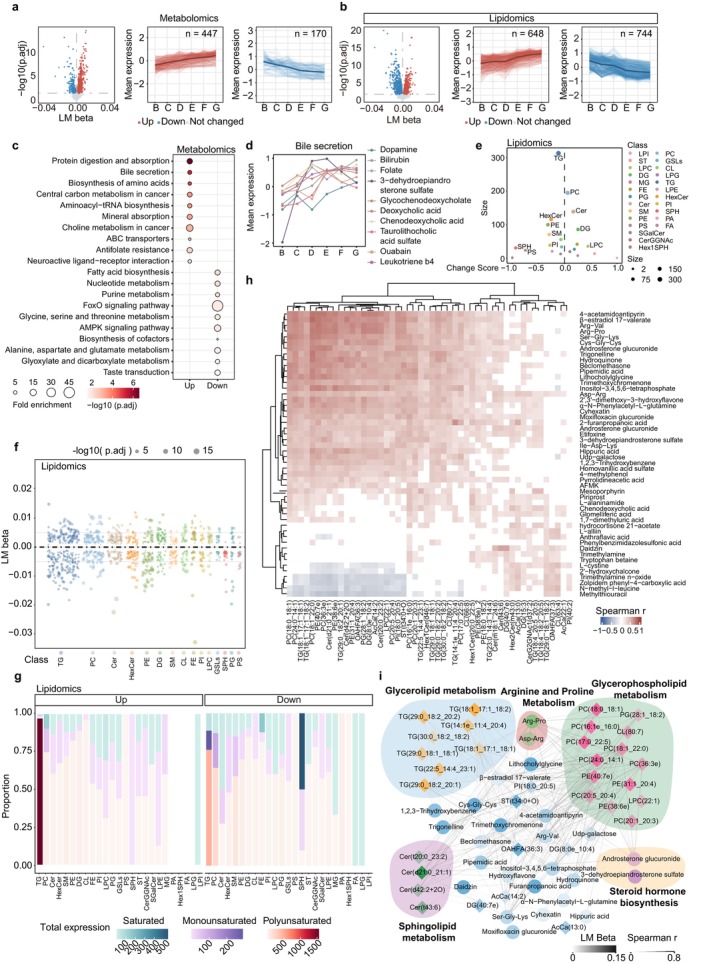
Linear changes in the metabolome and lipidome throughout the human lifespan. (a, b) Volcano plot (left) depicting the age‐related metabolites (a) and lipids (b) analyzed using a linear model. Statistical analyses were performed by *F*‐test, followed by BH correction. Line plots depicting scaled mean expression levels of metabolites with increasing (red, middle) or decreasing (blue, right) age. (c) KEGG metabolic pathway enriched by age linear related metabolites. The Fisher's exact test (one‐sided) followed by the BH‐corrected *p* value was used. (d) Scaled mean expression of enriched metabolites in the bile secretion pathway ((c) enriched). (e) Lollipop plot demonstrating the age‐related change score for the different lipid classes. Dot size reflects the quantity of metabolites associated with age. (f) Bubble plots depicting the LM beta of age‐related lipid classes. Values are shown as the LM beta from the linear model, dot size indicates significance. (g) Stacked bar chart graph depicting total expressions and proportions of unsaturation in age‐related lipid classes. The total expression indicates the sum of normalized intensities of all lipids within a given class. Stacked proportions denote the relative contribution of saturated (S, blue), monounsaturated (MU, purple), and polyunsaturated (PU, red) lipids to the total expression of that class. (h) Heatmap of Spearman correlation of top‐50 age‐increased metabolites and lipids. Only pair components with a *p* value < 0.05 are colored. (i) Spearman correlation networks for age‐increased metabolites and lipids with absolute correlation values above 0.5. The thickness and the transparency of the lines represent Spearman *r* and LM beta, respectively.

Given the continuous alterations in metabolic states across the lifespan, we subsequently investigated the age‐related linear changes in the metabolome and lipidome. Initially, we used a linear regression model to assess the DEMs that exhibited either an increase or decrease with advancing age, as well as showing relatively consistent patterns across both sexes (Figure 4a,b, Figure S5a). The altered components were concentrated in completely distinct modules (Figure 4c, Figure S5b). Similarly, metabolites showing linear age‐associated trends were stable across normalization methods (Figure S5c). Specifically, amino acids identified as elderly markers showed a steady linear increase across the lifespan, contrasting with their extremely low levels in newborns, which merits further investigation (Figures 2c and 4c). In addition, the progressive increase in bile acid–related metabolites may reflect age‐associated alterations in the metabolic pathways governing nutrient and lipid metabolism (Figure 4d, Figure S5d).

In the lipidome, various lipid types exhibit distinct expression trends during development (Figure 4e). SPH, particularly its saturated forms, which were notably high in newborns, showed a linear decline with age (Figure 4e,f, Figure S5e). In addition, TGs account for the highest proportion (Figure 4f). Intriguingly, despite the varying chain lengths, saturation levels, and expression trends among TGs, over 90% of upregulated TGs are polyunsaturated, while downregulated TGs exhibit a higher proportion and expression levels of saturated TGs (Figure 4g, Figure S5f). The plasma TG level is a crucial health indicator, correlated with an increased risk of age‐related diseases, such as coronary heart disease, atherosclerosis, myocardial infarction, and insulin resistance (Eichelmann et al. 2024). Therefore, understanding the variations in lipid saturation patterns may help in ultimately maintaining overall health.

We performed a Spearman correlation analysis to unravel the relationships between metabolites and lipids that exhibited linear increases with age (Figure 4h). The strong positive correlations between these metabolites and lipids may indicate that they share common functions and are situated within interconnected metabolic networks. To ascertain potential common regulatory relationships, we integrated the metabolites with lipids to construct a correlation network (Figure 4i). This network was based on the correlations between the top‐50 metabolites and top‐50 lipids with the highest linear change coefficients (*p*.adj < 0.05), and the correlations (Spearman *r* > 0.5) between 30 lipids and 28 metabolites are shown. The lipid pathway most strongly correlated with metabolites was glycerophospholipid metabolism. Conversely, TGs were the primary lipids correlated with metabolites in glycerolipid metabolism (Figure 4i). Overall, these findings highlight that the age‐related linear changes in metabolism across the lifespan are highly coordinated. Investigating these changes will provide insights into the linear alterations within the intricate metabolic landscape during the human development process.

### 2.5 Nonlinear Changes and Metabolic Peaks Associated With Aging

**FIGURE 5:**
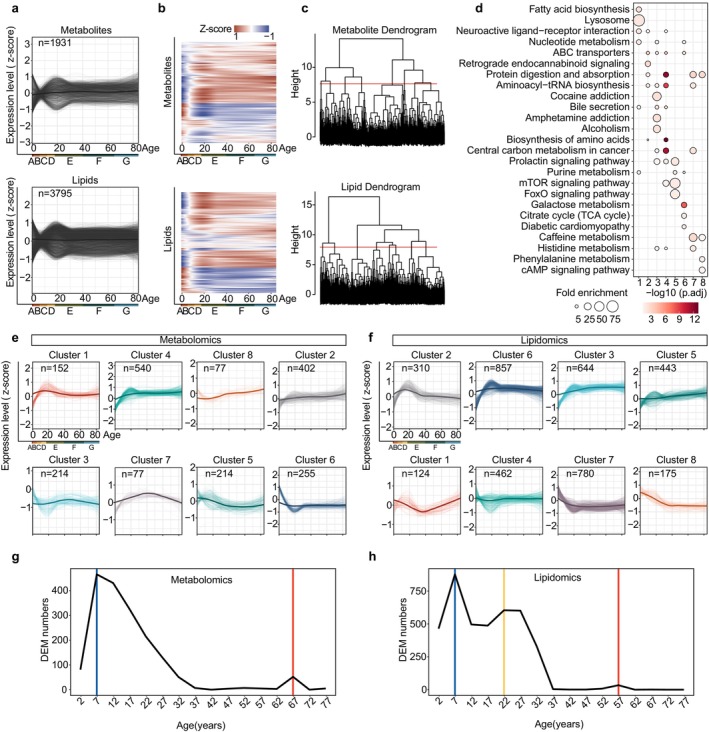
Clustering protein trajectories and sliding window analysis showing fluctuations in metabolites during aging. (a) Locally estimated scatterplot smoothing (LOESS) fitting trajectories with the aging process for metabolites (upper) and lipids (lower). (b) Unsupervised hierarchical clustering was performed to group metabolites (upper) and lipids (lower) with similar expression patterns. (c) Hierarchical clustering dendrogram intercepting and defining eight separate metabolites (upper) or lipids (lower) clusters. (d) The top‐4 KEGG metabolic pathways for each cluster in (c). Fisher's exact test (one‐sided), followed by the BH‐corrected *p* value was used. (e, f) LOESS trajectory plots of different metabolite (e) or lipid (f) clusters. The expression patterns of components across age are illustrated for all clusters, with thicker lines indicating the average trajectory for each cluster. (g, h) Differential Expression‐Sliding‐Window Analysis (DE‐SWAN) of different metabolites (g) and lipids (h) displaying the number of differentially presented features across ages, with an incremental window of 5 years.

While observing the linear variations in metabolic expression across ages, we concurrently noted several distinct nonlinear patterns of fluctuations among metabolites. Consequently, we employed locally estimated scatterplot smoothing (LOESS) to visualize the nonlinear changes in age‐related metabolites (Figure 5a). Additionally, unsupervised hierarchical clustering was applied to group the metabolites with similar expression patterns, thereby identifying the distinct trajectories of metabolic expression alterations with respect to age (Figure 5b). Within the eight clusters of metabolite analysis, each is enriched with specific biological pathways, indicating the occurrence of distinct, category‐specific changes in biological pathways across lifespan (Figure 5c,d). For example, Cluster 1, which peaks in expression around the age of 20, is represented by lysosome and fatty acid synthesis, while Cluster 7, peaking around 40 years, is predominantly characterized by caffeine metabolism (Figure 5d,e). Similarly, lipids were also clustered into distinct trajectories with notable differences in subclass proportions and saturation levels (Figure 5f, Figure S6a,b). In summary, many of the changes in the metabolome and lipidome across the lifespan are nonlinear. Clustering these changes by trajectory reveals distinct characteristics, demonstrating that the timing and duration of accumulation, or decline of certain components, may vary significantly across life. These findings enhance our understanding of key biological events, underscoring the dynamic regulatory patterns in biological pathways across the lifespan.

Next, we aimed to determine the ages at which the majority of metabolic expression changes occur, with the goal of identifying important metabolic turning points across the lifespan. DE‐SWAN (Differential Expression‐Sliding Window Analysis) is a specialized algorithm designed to analyze the differential expression of components across different age windows. Thus, we used DE‐SWAN with a 5‐year incremental window, spanning from the youngest to the oldest age groups. Several DE peaks were detected in the metabolome at ages 7 and 67 and in the lipidome at ages 7, 22, and 57; subsequently, we plotted the significant top metabolites corresponding to each peak (Figure 5g,h, Figure S6c). The DE‐SWAN‐identified age‐related peaks also remained robust under median‐ratio normalization (Figure S6d). It is noteworthy that the metabolome and lipidome reach a crest concurrently at the age of 7. However, in the later stages of life, the lipidome peaked earlier (age 57) than the metabolome (age 67) (Figure 5g,h). Additionally, the lipidome showed a unique peak at age 22, suggesting a rapid, more sensitive response to metabolic state changes than the metabolome, which may serve as a more agile reflection of alterations in an individual's health status, potentially acting as an early warning indicator. The limited overlap of metabolites and lipids at peak ages indicates that the developmental and aging processes in humans differ significantly across age stages (Figure S6e). Thus, the fluctuations in metabolism during the nonlinear process of aging are noteworthy.

### 2.6 Integrated Analysis With Other Datasets Reveals Key Metabolic Alterations in Aging

The terminal stage of lifespan—the period encompassing late adulthood, has emerged as a focal point of global concern. A deeper understanding of the processes associated with aging is required to achieve greater potential to enhance quality of life, as many of the diseases prevalent among the elderly are intimately tied to various metabolites in the body. Hence, we investigated the associations between metabolites (lipids) and clinical indicators during the transition from middle (Group F) to late adulthood (Group G) (Table S2).

**FIGURE 6:**
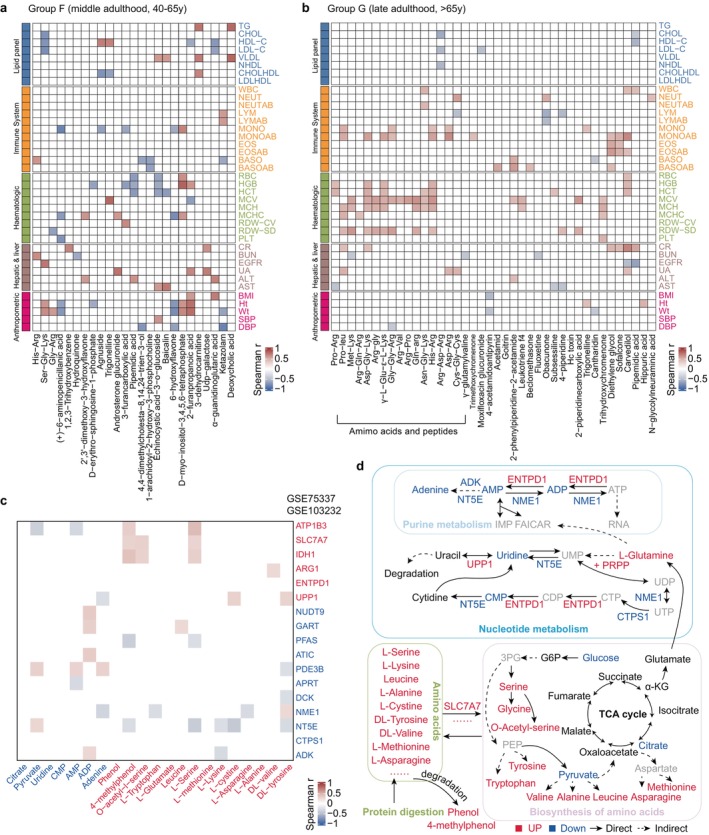
Linking metabolite profiles to other datasets in aging for health insight. (a) Spearman correlation analysis between clinical indicators and plasma marker metabolites in middle adulthood (Group F). (b) Spearman correlation between clinical indicators and plasma marker metabolites (top‐50 metabolites that generated correlations) in late adulthood (Group G). ALT, alanine aminotransferase; AST, aspartate aminotransferase; BASO, basophil percent; BASOAB, basophil absolute count; BMI, body mass index; BUN, blood urea nitrogen; CHOL, cholesterol; CHOLHDL, cholesterol to HDL ratio; CR, creatinine; DBP, diastolic blood pressure; EGFR, estimated glomerular filtration rate; EOS, eosinophil percent; EOSAB, eosinophil absolute count; HCT, hematocrit; HDL‐C, high density lipoprotein cholesterol; HGB, hemoglobin; Ht, height; LDL‐C, low density lipoprotein cholesterol; LDLHDL, LDL to HDL ratio; LYM, lymphocyte percent; LYMAB, lymphocyte absolute count; MCH, mean corpuscular hemoglobin; MCHC, mean corpuscular hemoglobin concentration; MCV, mean corpuscular volume; MONO, monocyte percent; MONOAB, monocyte absolute count; NEUT, neutrophil percent; NEUTAB, neutrophil absolute count; NHDL, non‐HDL; PLT, platelet; RBC, red blood cell count; RDW, red cell distribution width; SBP, systolic blood pressure; TG, triglyceride; UA, uric acid; WBC, white blood cell count; Wt, weight. (c) Spearman correlation between age‐increased metabolites and genes. Correlations are shown when the correlations were > 0. (d) Schema of metabolic pathways (purine and nucleotide metabolism, protein digestion, biosynthesis of amino acids and TCA cycle) with select metabolites and genes. Metabolites or genes which were significantly upregulated, downregulated and unchanged are colored in red, blue and black, respectively. Metabolites or genes colored in gray were not detected.

From middle age onward, certain amino acid peptides are increasingly expressed, with a notable accumulation identified by the elderly stage (Figure 6a,b). Amino acids are fundamental to protein synthesis, and are particularly crucial in erythropoiesis and hemoglobin synthesis, while also being significant for the stability of the cell membrane structure. Interestingly, these peptides show high positive correlations with red blood cell indices, particularly cell volume. On the one hand, this may help prevent conditions such as anemia; conversely, it could lead to structural instability during erythropoiesis, resulting in uneven sizes of newly formed red blood cells and increased blood viscosity. Additionally, the increase in the levels of amino acid peptides may promote inflammatory responses and elevate the risk of various diseases, such as diabetes (Ling et al. 2023). These physiological functions further underscore the importance of maintaining their balance for overall health. Currently, amino acid testing is largely absent from routine health examinations, indicating the need for increased monitoring of amino acid levels in the elderly.

We further observed multiple significant correlations between lipid profiles and clinical indicators. Notably, high‐density lipoprotein (HDL) (“good cholesterol”) is responsible for removing excess cholesterol; the lipids it carries are primarily phospholipids, particularly PC, with which it displays a strong positive correlation. Conversely, very low‐density lipoprotein (VLDL) (“bad cholesterol”) exhibits negative correlations with various lipid subclasses. These data underscore the intricate relationships between different lipid classes and blood lipid indicators, as the increases in different lipid subclasses may have varying impacts on health by influencing circulating levels of HDL or VLDL (Figure S7a,b).

Given that transcriptomics offers an alternative perspective into the metabolic networks at the level of the metabolic enzymes, we performed an integrated analysis of metabolomic and published transcriptomic data (Peters et al. 2015) to gain deeper insights into the metabolic trends in the elderly. Consistent with our hypothesis, the accumulation of amino acids in the elderly was supported by our identification of a positive correlation in transcriptomics and metabolomics (Figure 6c). Specifically, the accumulation of various amino acids produced from protein digestion and generated using TCA cycle metabolites as raw materials was observed, whereas the expression of these raw materials declined (Figure 6d). Furthermore, glutamine contributes to nucleotide synthesis, while the levels of metabolites and enzymes associated with nucleotide and purine metabolism decline. This suggests that in the elderly, amino acid production is high, but utilization is either untimely or sufficient. Even worse, the accumulation of substantial amino acids leads to an increase in phenolic degradation products, inflicting detrimental effects on health. It is worth noting that, the correlations between metabolomic features and transcriptomic data were derived from different populations, and thus should be regarded as only exploratory: the analysis results require validation in future studies using matched cohorts.

### 2.7 Measuring Biological Age Using Multi‐Dimensional Aging Clocks

**FIGURE 7:**
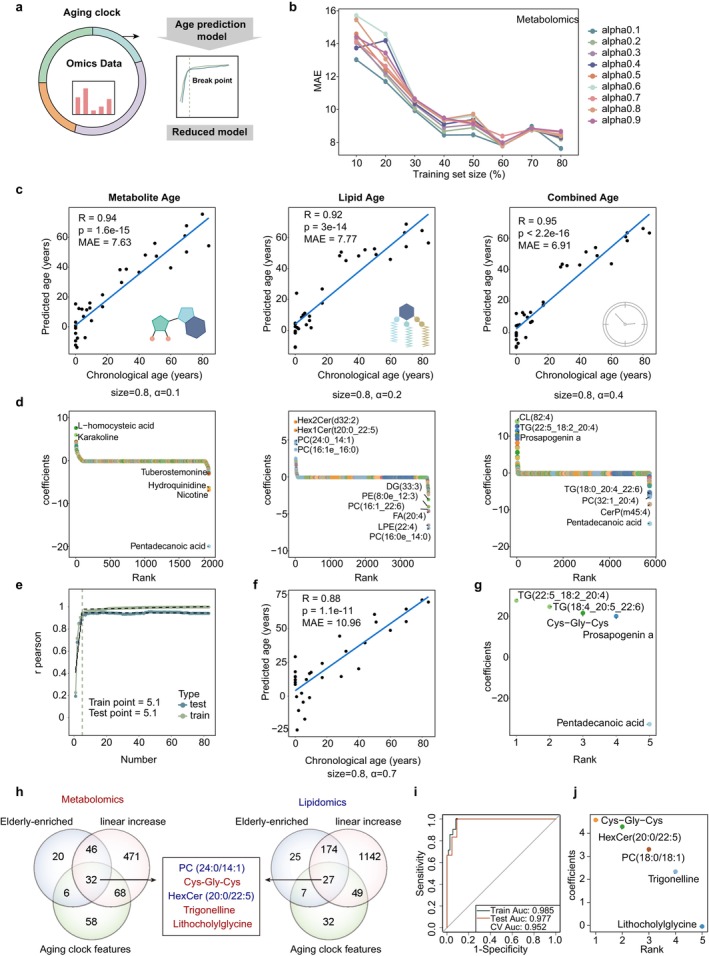
Aging clock built from metabolomics and lipidomics. (a) Schematic showing the establishment of the Age prediction model. (b) Line plot showing the mean absolute error (MAE) for the metabolic aging clock with different alpha parameters and different training set sizes. (c) The linear relationship between the predicted ages from the age predictors and chronological age is shown. The Pearson's correlation coefficients (*R*) and MAE are presented. The prediction capabilities of different aging clock models (metabolite, lipid, and metabolite–lipid combined aging clock models) are shown. (d) Dot plots illustrating the components of different aging clock models (metabolite, lipid, and metabolite–lipid combined aging clock models). Dots are colored by substance classes, as shown in Extended Data. (e) Dashed lines represent a broken‐stick regression, marking the break point between the number of metabolites and prediction accuracy. The Pearson correlation (*R*) represents the relationship between the predicted and chronological ages. (f) The linear relationship between the predicted ages and chronological age in the reduced metabolite–lipid combined model. (g) Dot plots illustrating the components of the reduced combined model. Dots are colored by substance classes, as in Extended Data. (h) Venn diagrams showing the overlap among three feature sets: metabolites or lipids elevated in the elderly, linearly correlated with age, and selected by the Elastic Net model. The left panel represents the metabolites, and the right panel represents the lipids. (i) Receiver operating characteristic (ROC) curve of the final panel discriminating the elderly (≥ 65 years) from younger (< 40 years) individuals. (j) Dot plots illustrating the components of the final predictive panel.

In the same way, given the focus on aging and the complex, dynamic nature of the aging process, we developed a “metabolite–lipid clock” to predict chronological age based on our data. Using the ElasticNet model, we constructed three clocks—a “metabolite clock,” a “lipid clock,” and a “combined clock”—across the age cohort to assess biological age through the integration of multi‐dimensional information (Figure 7a,b). A total of 1931 metabolites and 3795 lipids were used to build these models, ultimately constructing a metabolite clock comprising 107 metabolites, a lipid clock comprising 68 lipids, and a combined clock consisting of 53 components (Figure 7c,d). The three aging clock models were all maintained within a mean absolute error (MAE) of 8 years. Furthermore, the combined clock achieved the lowest MAE and the highest correlation, thereby improving the precision of biological age estimation by capturing changes across different aging trajectories (Figure 7c,d). In addition, we performed an additional validation of our metabolic aging clock using an independent cohort of 12 individuals not included in our dataset (Figure S8a). The model demonstrated consistent predictive accuracy in this validation cohort, supporting its preliminary robustness.

To enhance the practicality and ease of use in clinical applications, we subsequently applied broken‐stick regression to identify the breakpoint that balances the number of metabolites and prediction accuracy, thereby developing a reduced age clock model with fewer components while maintaining precise age predictions (Figure 7f). This clock included only five components, but nevertheless showed a strong correlation (*r* = 0.88) and robust predictive performance (Figure 7g,h). Similarly, we constructed reduced metabolite and lipid clocks, containing only nine metabolites and seven lipids, respectively, both of which accurately predicted age (Figure S8b–d). To identify aging‐related biomarkers, we applied three complementary selection strategies: (1) features significantly upregulated in the elderly (≥ 65 years), (2) features showing a linear increase across the lifespan, and (3) features with high predictive weight in our Elastic Net age model. The intersection yielded 32 metabolites and 27 lipids (Figure 7h), from which we constructed a concise five‐marker panel. The expression level of this panel positively correlated with the degree of aging. Further, this panel was validated by 10‐fold cross‐validation, achieving a mean area under the curve (AUC) of 0.952 (Figure 7i,j). We subsequently identified aging‐related biomarkers that accurately reflect age‐associated metabolic features.

Overall, our findings reveal the potential of metabolite component‐based aging clocks as valuable tools for advancing aging research, with particular attention to the combined clock for its high precision and minimal component breakpoint.

## 3 Discussion

Metabolic changes across the human lifespan are critical to understanding aging and developing effective interventions for age‐related diseases. In the present study, we provide a comprehensive analysis of the dynamic changes in metabolic profiles from infancy to old age. Our findings thus offer novel insights into the metabolic changes that occur during key life stages and highlight the biological processes driving these transitions.

Metabolomics offers a powerful tool for investigating physiological changes, capturing rapid metabolic shifts with high sensitivity. However, current methods commonly prioritize hydrophilic (polar) compounds due to the reliance on sample preparation techniques like hydrophilic interaction liquid chromatography (HILIC) (Tian et al. 2022). This approach limits the exploration of hydrophobic (nonpolar) compounds, such as lipids, which are also critical for understanding metabolism across different life stages. Our study addresses this limitation by conducting broad, full‐spectrum metabolomic and lipidomic analyses in plasma across a wide age range, thereby providing a more comprehensive view of metabolic changes throughout the human lifespan. Nevertheless, as this study only analyzed plasma samples, the tissue of origin of specific metabolites and lipids cannot be determined. Future studies integrating tissue‐resolved datasets will be valuable for delineating organ‐specific metabolic changes.

One key observation of our study is the unique metabolic profile of newborns, characterized by a high metabolic rate and distinct SPH features. Prior research (Dei Cas et al. 2020) has highlighted the importance of SM and its derivative, SPH, in breast milk for supporting neonatal growth and brain development. Our findings indicate a significant elevation of SPH bases in newborns, whereas SM and Cer levels remain low. Previous evidence (Lee et al. 2023) has demonstrated that sphingolipid metabolites regulate immune cells, while our data indicate that SPH can induce apoptosis and modulate immune receptor expression. These findings point to SPH as a potential link between lipid metabolism and the immune system, and suggest that elevated SPH may help to prevent excessive immune activation during the newborn period. On the other hand, since cord blood was used for newborns and peripheral blood for other groups, potential matrix heterogeneity should be considered when interpreting metabolite differences across ages.

Our data suggest that the accumulation of amino acids in the elderly, may reflect heightened metabolic activity; however, this accumulation could also indicate a pathological imbalance in which amino acid catabolism lags behind synthesis, leading to the build‐up of potentially toxic metabolites. Alternatively, this may represent a compensatory but dysregulated response to age‐related anabolic resistance or tissue wasting, reflecting an adaptive attempt to sustain protein turnover despite diminished catabolism capacity. In our study, we identified a characteristic aging‐associated metabolic pattern marked by amino acid enrichment, bile acid accumulation, and lipid imbalance. Consistent with this, we established a five‐marker panel comprising metabolites and lipids—Trigonelline, Lithocholylglycine, Cys–Gly–Cys, PC (18:0/18:1) and HexCer (20:0/22:5). Trigonelline is a NAD^+^ precursor supporting mitochondrial function and metabolic health (Membrez et al. 2024); Lithocholylglycine is a bile acid derivative implicated in systemic metabolism and cognitive function (Qu et al. 2025); Cys–Gly–Cys is linked to glutathione synthesis and redox control (Maher 2005); HexCer and other sphingolipids regulate cellular senescence and apoptosis (Trayssac et al. 2018); and PC species are involved in membrane remodeling and longevity regulation (Hornburg et al. 2023; Kim et al. 2019). Overall, our findings are consistent with established hallmarks of aging across multiple metabolic pathways, while recognizing that these associations are correlative and require further study to determine causality.

To further explore these metabolic trends, we applied linear and nonlinear models, along with sliding window analysis, allowing us to identify key metabolic turning points across the lifespan. A recent report (Shen et al. 2024) has noted nonlinear shifts across multi‐omics data, particularly in alcohol and caffeine metabolism around the ages 40 and 60, within a cluster. Our analysis supports these findings, showing similar shifts in Cluster 3, in which key metabolites peak at these ages. Additionally, we identified a distinct group of caffeine metabolites in Cluster 7, which peak around the age of 40, suggesting that certain metabolite classes follow unique trajectories that diverge with age. Peaks identified at 22 and 67 years align closely with previous findings (Lehallier et al. 2019; Li, Xiong, et al. 2023; Shen et al. 2024), reinforcing the idea that young adults and the elderly are marked by significant metabolic shifts. However, our analysis at age 7 captured unique metabolite–lipid peaks, that may be influenced by differences in analytical windows or techniques. Furthermore, we detected an early peak in lipid metabolism in the elderly, suggesting that lipid responses to aging may occur earlier and more rapidly than traditionally assumed.

The concept of an aging clock has emerged as an important tool for understanding biological aging. Over the past decade, epigenetic clocks (Hannum et al. 2013; Horvath 2013) have attracted significant attention, leading to the development of increasingly accurate clocks (Argentieri et al. 2024; Fong et al. 2024; Hwangbo et al. 2022; Janssens et al. 2023; Lehallier et al. 2019; Li, Xiong, et al. 2023; Meyer and Schumacher 2024). A recent large‐scale review (Bao et al. 2023) summarized the advances in aging clock research from various perspectives, highlighting the potential of combining multiple clock models to improve accuracy and applicability. To contextualize our model's performance, we compared it with established aging clocks and found it achieved comparable accuracy to those from larger cohorts (Figure S7d). Meanwhile, our metabolic clock reflects dynamic, real‐time physiological aging, making it suitable for monitoring lifestyle, dietary, or pharmacological interventions. The reduced model requires fewer measurable components, enhancing usability, while its minimally invasive and cost‐effective nature increases its translational potential. Future research can use this framework to examine the connection between metabolic aging and health outcomes and explore interventions to slow aging or prevent age‐related diseases.

Several limitations of this study should be considered. First, the cohort size was relatively small (*n* = 136) and unevenly distributed across age groups which may limit the generalizability of the findings. Second, although participants spanned a wide age range, the study design was cross‐sectional; longitudinal cohorts will be required to capture more robustly temporal dynamics. Third, although we adjusted for sex, other key covariates such as BMI, dietary habits, physical activity, and subclinical conditions were not uniformly available. Future studies leveraging more comprehensively characterized cohorts will be crucial for systematically adjusting for a broader array of confounders. Finally, the multi‐omics aging clock model has not yet been validated in individuals with clinical information; this lack of validation in an independent external cohort represents a major limitation, and the reduced model may be particularly susceptible to overfitting. Future studies with larger, longitudinal, and externally validated cohorts, including comprehensive clinical and lifestyle information, will be essential to further validate the robustness and translational value of our findings.

In conclusion, this study provides valuable novel insights into the metabolic changes that occur throughout the human lifespan. By identifying linear and nonlinear trajectories of metabolism, and linking these changes to key biological processes, we were able to establish a framework for future interventions aimed at improving health across the human lifespan. Our findings suggest that targeted metabolic interventions, informed by aging clock models and comprehensive metabolomics data, may thus offer promising strategies to prevent age‐related metabolic diseases and promote healthier aging.

## 4 Conclusions

In summary, the present study makes a significant contribution to the field of metabolic research by providing an integrated view of how metabolism evolves across the human lifespan. These insights will not only advance basic scientific understanding but also have the potential to drive therapeutic innovation in aging, metabolic disorders, and precision medicine.

## 5 Methods

### 5.1 Human Samples

Samples of cord blood (CB) and peripheral blood (PB) were obtained from the First Affiliated Hospital of the University of Science and Technology of China. This study was approved by the Ethics Committee of the University of Science and Technology of China (approval no. 2022‐ky022; no. 2022‐ky062). Clinical characteristics are listed in Table S1. All adult blood samples were collected after overnight fasting (≥ 8 h) between 7:00 and 9:30 a.m. Participants were requested to avoid strenuous exercise and alcohol consumption within the 24 h prior to sample collection. Information on medication use and other clinical variables was reviewed to exclude individuals with acute or chronic diseases that could markedly affect metabolism. Fresh whole blood samples were collected in ethylenediaminetetraacetic acid (EDTA)‐containing tubes, and centrifuged to obtain plasma supernatant. Blood was processed within 1 h of sampling, and the collected plasma was stored at −80°C until metabolite extraction.

### 5.2 Sample Preparation

For metabolite extraction, 100 μL of plasma was mixed with 400 μL of ice‐cold methanol/acetonitrile (1:1, v/v) to precipitate proteins. After a 20‐min centrifugation at 14,000 *g* and 4°C, the supernatant was vacuum dried. Samples were then reconstituted in 100 μL acetonitrile/water (1:1, v/v) and centrifuged again prior to LC–MS.

The MTBE method was used for lipid extraction. Briefly, 200 μL of water was added to the sample and then vortexed. Afterward, 240 μL of precooled methanol was added, followed by vortexing for 30 s. Next, 800 μL of MTBE was added, after which the mixture underwent a 20‐min ultrasound at 4°C, followed by incubation at room temperature for 30 min. Following centrifugation of the solution at 14,000 *g* for 15 min at 10°C, the upper organic layer was collected and dried under nitrogen.

### 5.3 Untargeted Metabolomics Analysis

The analysis was conducted using an UHPLC system (Agilent 1290 Infinity) paired with a quadrupole time‐of‐flight mass spectrometer (AB Sciex TripleTOF 6600). A 2.1 mm × 100 mm ACQUIY UPLC BEH Amide 1.7 μm column was used for HILIC separation. In both ESI positive and negative modes, the mobile phases were 25 mM ammonium acetate/ammonium hydroxide (A) and acetonitrile (B). The gradient began at 95% B, and was gradually decreased to 65% over 6.5 min, then down to 40% in 1 min, held for 1 min, and returned to 95% B for re‐equilibration. MS scan conditions included a range of m/z 60–1000 Da, with a 0.20 s accumulation time, while auto MS/MS scans covered m/z 25–1000 Da with a 0.05 s accumulation. Parameters for auto MS/MS used IDA with a fixed collision energy of 35 V (±15 eV) and declustering potentials (DP) of 60 V (+) and 60 V (−).

For eliminating contaminants in samples, blank samples (e.g., solvent blanks) were included in each batch to monitor potential background contamination, with detected signals consistently lower in intensity than those in biological samples to minimize their impact on differential analysis. Low‐intensity signals below predefined thresholds were systematically filtered out during peak extraction and alignment to reduce background noise. Features frequently detected at high intensity in blank samples, such as reagent additives or carryover contaminants, were flagged and excluded from downstream analysis.

### 5.4 Untargeted Lipidomics Analysis

Reverse phase chromatography was applied for LC separation with a CSH C18 column (1.7 μm, 2.1 mm × 100 mm, Waters). Lipid extracts were re‐dissolved in 200 μL of 90% isopropanol/acetonitrile, centrifuged at 14,000 *g* for 15 min, and 3 μL of sample was injected. Solvent A consisted of acetonitrile–water (6:4, v/v) with 0.1% formic acid and 0.1 mM ammonium formate, and solvent B was acetonitrile–isopropanol (1:9, v/v) with the same additives. The flow rate started at 30% B, increased to 100% over 23 min, and re‐equilibrated. Mass spectra were acquired using Q‐Exactive Plus in the positive and negative modes, respectively. ESI settings were as follows: 300°C source temperature, 350°C capillary temperature, 3000 V ion spray voltage, and scan range of m/z 200–1800. For lipid identification, we used LipidSearch (v.4.0), a widely recognized lipidomics software. Lipid molecules and internal standards were processed using peak recognition, peak extraction, and MS/MS‐based lipid identification. The main parameters applied were as follows: precursor tolerance of 5 ppm, product tolerance of 5 ppm, and product ion threshold of 5%. These stringent settings ensured accurate annotation while minimizing redundancy.

### 5.5 Data Processing

Raw MS data were converted to MzXML format using ProteoWizard MSConvert (v.3.0.6428) (Chambers et al. 2012) and processed in XCMS online (v.3.7.1) (Benton et al. 2008). Peak picking was performed with centWave (m/z = 10 ppm, peakwidth = c (10, 60), prefilter = c (10, 100)), and peak grouping with bw = 5, mzwid = 0.025, minfrac = 0.5. Isotopes and adducts were annotated using CAMERA (Kuhl et al. 2012). Only variables with more than 50% nonzero values in at least one group were retained. Metabolites were first screened against a credible spectral database (this database integrates over 6000 compounds from our in‐house database with entries from four major public databases—MassBank, METLIN, MoNA, and the commercial version of HMDB), maintaining a strict mass accuracy threshold of ≤ 10 ppm to minimize false positives. Identification was further refined by comparing fragmentation patterns with spectral databases, ensuring a high‐confidence annotation with a similarity score > 0.6, thereby improving accuracy beyond simple precursor mass matching (Schrimpe‐Rutledge et al. 2016). All identified metabolites meet at least MSI Level 2 standards. To ensure data quality, all biological samples were randomized prior to LC–MS injection. In addition, pooled QC samples were injected at regular intervals (every 3 samples) throughout the analytical sequence to monitor instrument stability and signal drift. We applied QC‐based Support Vector Regression (QC‐SVR) for batch correction, a well‐established approach in large‐scale metabolomics studies (Dunn et al. 2011; Shen et al. 2016). Additionally, we applied total sum normalization (TSN) after QC correction to improve comparability across samples. Data quality was comprehensively evaluated: TIC overlap of QC samples confirmed consistent chromatographic performance; PCA and Pearson correlation analyses revealed tight QC clustering and correlations > 0.9; Hotelling's *T* ^2^ test indicated all QC samples fell within the 99% confidence interval, and the proportion of metabolites and lipids with a coefficient of variation of < 30% in QC samples was maintained at > 80%. Specifically, the final mean/median CV values are 20.67%/20.072% for metabolomics and 18.532%/11.469% for lipidomics. The normalized intensity was obtained by dividing the raw intensity by the sum of each sample. To assess the robustness of the results, the raw intensity was further divided by the median of each sample for normalization in key findings.

### 5.6 PCA and PVCA for Metabolomics and Lipidomics Data

The normalized abundance matrices of log2‐transformed metabolites or lipids were used for PCA and PVCA, using the prcomp and pvcaBatchAssess functions under R stat (v.4.1.2) and pvca (v.1.34.0) (Li et al. 2009) packages. The results were visualized using the factoextra (v.1.0.7) and ggplot2 (v.3.5.1) packages.

### 5.7 Differential Expression (DE) Analysis of Metabolic or Lipid Profiles

Statistical differences in the metabolites or lipids were analyzed using the R stats (v.4.1.2) package with Student's *t*‐test (two‐sided) and adjusted using the Benjamini–Hochberg (BH) multiple comparisons test. For comparisons between age groups, adjusted *p* values < 0.05 were considered statistically significant. The UpSetR (v.1.4.0) (Conway et al. 2017) and VennDiagram (v.1.7.3) (Chen and Boutros 2011) packages were used to identify intersections between different sets. Heatmaps of metabolite or lipid expression were generated using the Complexheatmap (v.2.10.0) (Gu et al. 2016) package. The age‐related change scores were calculated based on the previously reported method (Li, Xiong, et al. 2023).

### 5.8 KEGG Pathway Enrichment

To annotate the pathways of the DEMs, we downloaded the KEGG metabolic pathways and associated metabolites from the KEGG API (https://www.kegg.jp/kegg/rest/keggapi.html). KEGG pathway enrichment was subsequently performed using the R clusterProfiler (v.4.2.2) (Wu et al. 2021) package and significant pathways were obtained with adjusted *p* value < 0.05 and minGSSize = 2.

### 5.9 Linear Age Changes in Metabolome and Lipidome

To characterize age‐related metabolic and lipid changes, the following model was implemented, with sex included as a covariate to adjust for potential confounding:

$$\text{Expression}\sim \alpha +\beta_{1}\mathrm{Age}+\beta_{2}\text{Gender}+\varepsilon$$

where “*α*” represents the *y*‐intercept, “*β*” represents the slope, and “*ε*” represents residual error. Log10 transformed normalized matrices of metabolites or lipids were used as expression values. The linear model and Type II sums of squares were generated using the LM and Anova functions in the R stat (v.4.1.2) and car (v.3.1.2) (Weisberg and Fox 2010) packages, respectively. Significant changes were classified by adjusted *p* values < 0.05.

### 5.10 Nonlinear Age Changes in Metabolome and Lipidome

Nonlinear patterns of metabolite and lipid expression with age were determined using LOESS models, implemented using the loess function from the R stats (v.4.1.2) package. The predicted age‐related trajectories of metabolite and lipid expression were subsequently hierarchically clustered into eight groups using the hclust function in the R stats (v.4.1.2) package.

### 5.11 DE‐SWAN Analysis for Metabolomics and Lipidomics Data

The DE‐SWAN (v.0.0.0.9001) (Lehallier et al. 2019) package in R was used to identify transient changes in metabolite and lipid expression across the lifespan, with sex included as a covariate to adjust for potential confounding. Comparisons were made in 5‐year intervals across a 20‐year window. Significant changes were determined using BH‐adjusted *p* values < 0.05 and “*β*” thresholds.

### 5.12 Correlation Analysis

Correlations between the top‐50 age‐increased metabolites and lipids were calculated through Spearman's correlation conducted using the cor function in the R stat (v.4.1.2) package. In addition, we analyzed the correlation between components and clinical information of the samples. The bulk RNA‐seq count matrices of GSE75337 and GSE103232 (Aramillo Irizar et al. 2018) were downloaded and normalized using the DEseq2 (v.1.34.0) (Love et al. 2014) package to perform the same correlation analysis with our metabolite data. The *p* value of the correlation coefficient was calculated by the *t*‐test formula. Network visualization was performed using Cytoscape (v.3.10.2) (Shannon et al. 2003) software.

### 5.13 Prediction of Human Biological Age Using Metabolomic and Lipidomic Data

To evaluate the ability of our metabolomics and lipidomics data to predict organismal age, we constructed models using different training set sizes and alpha values of 0.1–0.9 with the glmnet (v. 4.1.8) (Friedman et al. 2010) package. Elastic Net regression, which combines the advantages of an L1‐norm (Lasso) regression and an L2‐norm (Ridge) regression model was applied, ensuring that feature selection is robust (Friedman et al. 2010). Lambda values were optimized by 10‐fold cross‐validation. At least 50% of the dataset was used for training to balance model learning and generalization, with the remaining samples reserved for validation. The model with the smallest MAE was selected as the final model.

To determine whether a subset of metabolites or lipids could also be used to predict age with comparable accuracy, we developed a reduced model. First, we obtained the metabolites and lipids included in the initial age‐clock model. These features were ranked based on the absolute values of their correlation coefficient, with higher absolute values indicating a greater contribution to the model. A series of elastic net models were constructed by progressively incorporating top‐ranked features (alpha value: 0.1–0.9, train set size: 80%). Specifically, models were built using the top 2, top 3, top 4 features, and so on, systematically evaluating how model performance changes with feature number. The predictive accuracy of these models was then calculated, and the segmented (v. 1.6.1) (Fasola et al. 2018) package was used to identify the breakpoint between the number of metabolites and prediction accuracy.

### 5.14 Identification of Aging‐Related Biomarkers

To identify robust metabolic features associated with aging, the overlapping components (32 metabolites and 27 lipids) were used to train a logistic regression model with glmnet (v4.1.8). Model performance was evaluated by 10‐fold cross‐validation implemented via the caret (v6.0.93). Receiver operating characteristic (ROC) analysis was conducted using pROC (v1.18.0) (Robin et al. 2011).

### 5.15 Isolation and Culture of Cord Blood Mononuclear Cells (CBMCs)

CBMCs were isolated from cord blood by Ficoll cell separation solution (TBDscience, Cat# LTS1077) density gradient centrifugation. After isolation, CBMCs were cultured in RPMI 1640 (Biosharp, Cat# BL303A) supplemented with 10% FBS (Sigma, Cat# F7524) and 1 × penicillin–streptomycin–amphotericin (Biosharp, Cat# BL142A). For metabolite treatment, CBMCs were resuspended in RPMI 1640 complete medium plus SPH (10 μM, MCE, Cat# HY‐W019838) for 24 h.

### 5.16 Flow Cytometry

CBMCs were labeled with conjugated antibodies for 30 min at 4°C and subsequently analyzed by flow cytometry. IgG isotype controls were used for all staining procedures. CD45 (Biolegend, Cat# 304036), CD3 (Biolegend, Cat# 300420), CD19 (Biolegend, Cat# 302243), CD56 (Biolegend, Cat# 362552), CD4 (Biolegend, Cat# 357417), CD8 (Biolegend, Cat# 344713), CD69 (Biolegend, Cat# 310904), CD38 (Biolegend, Cat# 303506), DNAM‐1 (Biolegend, Cat# 338314), PD1 (Biolegend, Cat# 621610), TIGIT (Biolegend, Cat# 372704), and Tim3 (Biolegend, Cat# 345022) antibodies were used. For apoptosis analysis, cells were stained with Annexin V/PI (Elabscience, Cat# E‐CK‐A217) for 15 min and analyzed by flow cytometry. Flow cytometry was performed on a Beckman Coulter CytoFLEX flow cytometer, and the data were analyzed using FlowJo software. The cell gating strategies were shown in Figure S3a.

### 5.17 Quantification and Statistical Analysis

R statistical software v4.1.2 was used for all statistical analyses. Statistical methods are described in the figure legends as appropriate.

## Author Contributions

F.N. conceived and conducted the project. F.N., X.L., and T.L. designed the experiments. X.L. and T.L. performed the data analysis. R.Z. and X.H. collected human samples and clinical information. B.H. and M.Z. provided comments and suggestions. X.L., T.L., and F.N. wrote the manuscript.

## Funding

This work was supported by the Strategic Priority Research Program of the Chinese Academy of Sciences (XDB0940000), the Natural Science Foundation of China (32070916, 82370159), the Extension Grant of the Anhui Provincial Distinguished Young Scholars Science Foundation (2408085JX012), and the Research Funds of Center for Advanced Interdisciplinary Science and Biomedicine of IHM (QYPY20220007).

## Conflicts of Interest

The authors declare no conflicts of interest.

## Supporting information


**Data S1:** acel70316‐sup‐0001‐Supinfo.docx.

## Data Availability

The metabolomic and lipidomic data used in this study have been submitted to OMIX (https://ngdc.cncb.ac.cn/omix/), and can be accessed using accession ID OMIX007740. The information of each donor is provided in Table S1. Clinical information used for correlation analysis is provided in Table S2. Codes for data analysis are available at https://github.com/Tility/lifespan_metabolite.
