# Supplementary material for: A Global Metabolomic and Lipidomic Landscape of Human Plasma Across the Lifespan
Source: Aging Cell. 2025 Dec 6;25(1):e70316. doi: 10.1111/acel.70316 (PMC12741190; doi:10.1111/acel.70316)
Supplement: Supplementary file 1 — Data S1: acel70316‐sup‐0001‐Supinfo.docx. [file ACEL-25-e70316-s001.docx]

**Supplementary Information**

**A global metabolomic and lipidomic landscape of human plasma across the lifespan**

Xinru Liu^1,2^, Tingting Liang^1,2^, Rui Zhao^1,2^, Mingming Zhu^1,2^, Beibei Huang^3^, Xiaobi Huang^4^, and Fang Ni^1,2*^

Xinru Liu and Tingting Liang contributed equally to this work

Correspondence to: fangni@ustc.edu.cn

**This file includes:**

Figures. S1 to S8

Tables S1 to S2


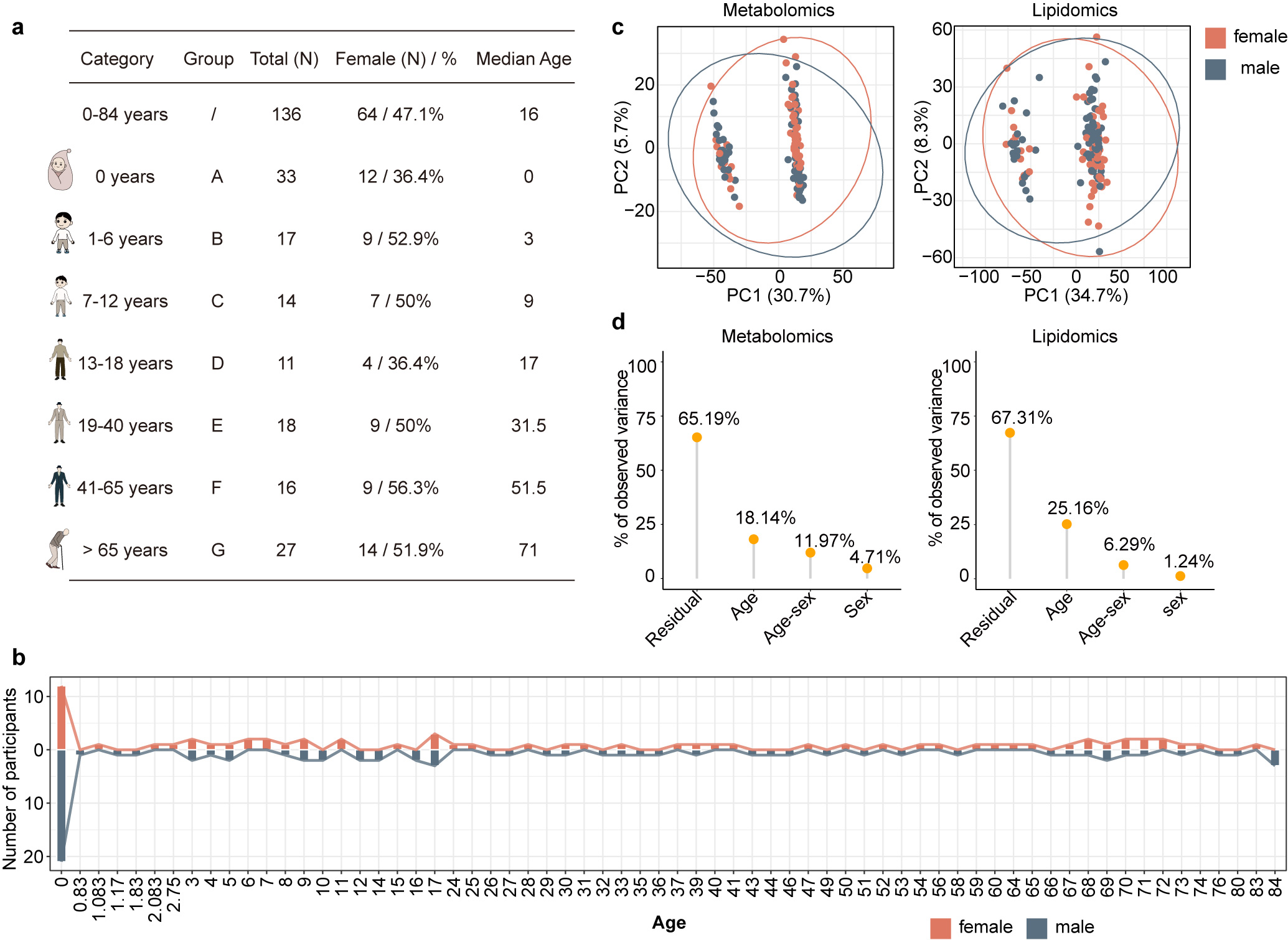


**Figure. S1. Quality assessments for metabolomic and lipidomic data, related to Figure 1. a** Summary of participant information, including group size, sex composition, and median age. **b** Distribution of participants by different stage and gender. The x-axis represents age, and the y-axis represents the number of participants included in the study. **c** PCA score plots for metabolomics (left) and lipidomics (right) on sex, including all samples across the total lifespan. Each sample is colored by sex information. **d** Visualization of the principal variance component analysis (PVCA) after excluding the newborn group, displaying the variance in metabolite and lipid expression explained by residuals (technical and biological noise) and experimental factors (sex, age, and age–sex).

**
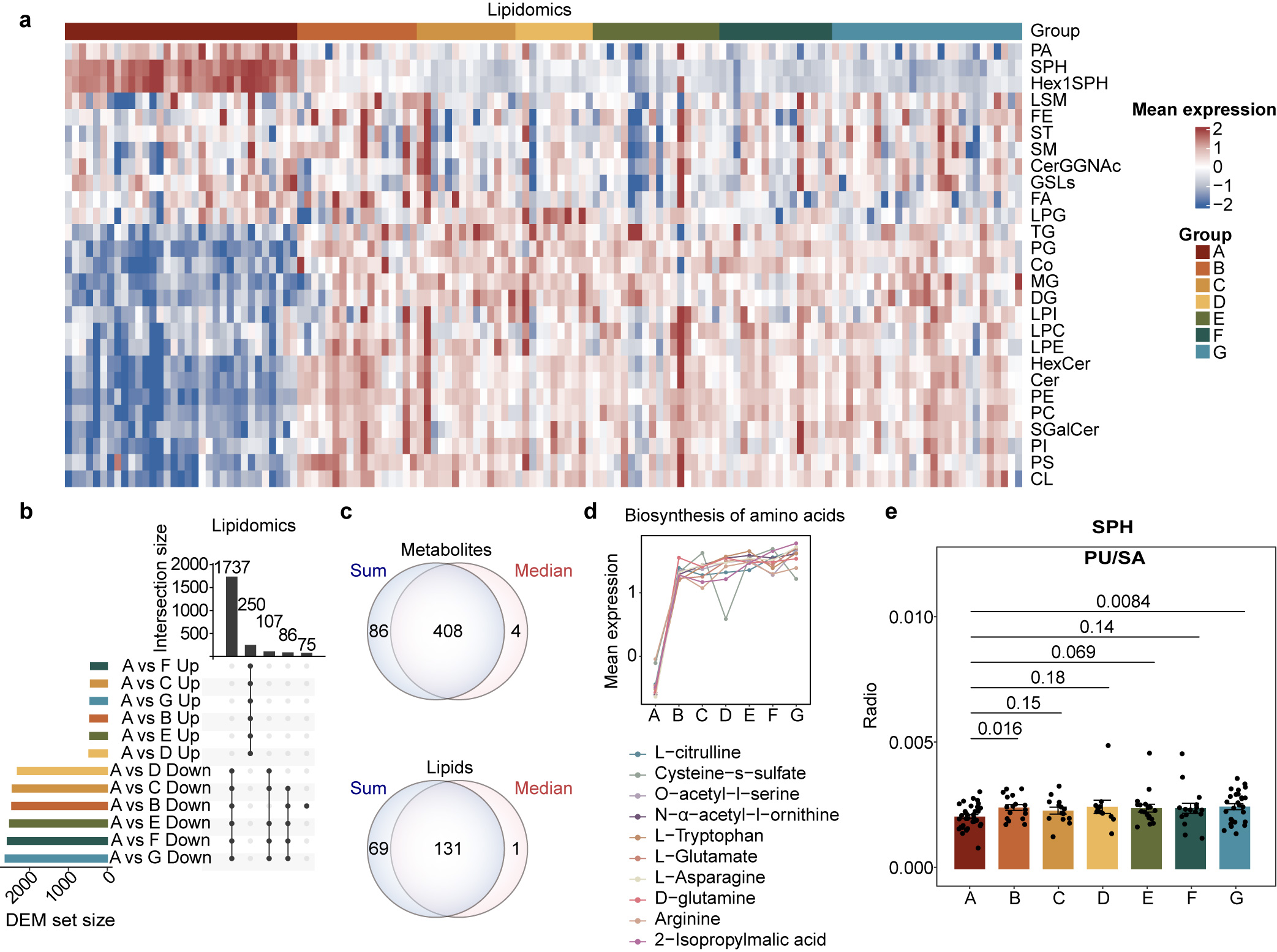
** **Figure. S2. Significant perturbation in the plasma metabolome of newborns, related to Figure 2**. **a** Heatmap of the relative abundances of metabolites and lipids across different age groups. Color bars indicate the age groups. **b** UpSet plot showing the number of DEMs (lipids) between newborn and other age groups. Statistical analyses were performed using two-sided Student’s t-test followed by Benjamini–Hochberg (BH) correction, the significantly changed metabolites were determined by a BH-corrected P value < 0.05. **c** Venn plots depicting the overlap of newborn markers identified by total-sum and median normalization for metabolites (left) and lipids (right). One-sided Fisher’s exact test, ***P < 0.0001. **d** Scaled mean expression of metabolites in KEGG up pathway (Biosynthesis of amino acids). **e** Bar graphs showing the ratios of the concentrations of polyunsaturated SPHs versus saturated SPHs in different age groups, for which each dot represents one biological replicate and presented as the mean ± s.e.m. Statistical significance was determined by two-tailed unpaired t-test.
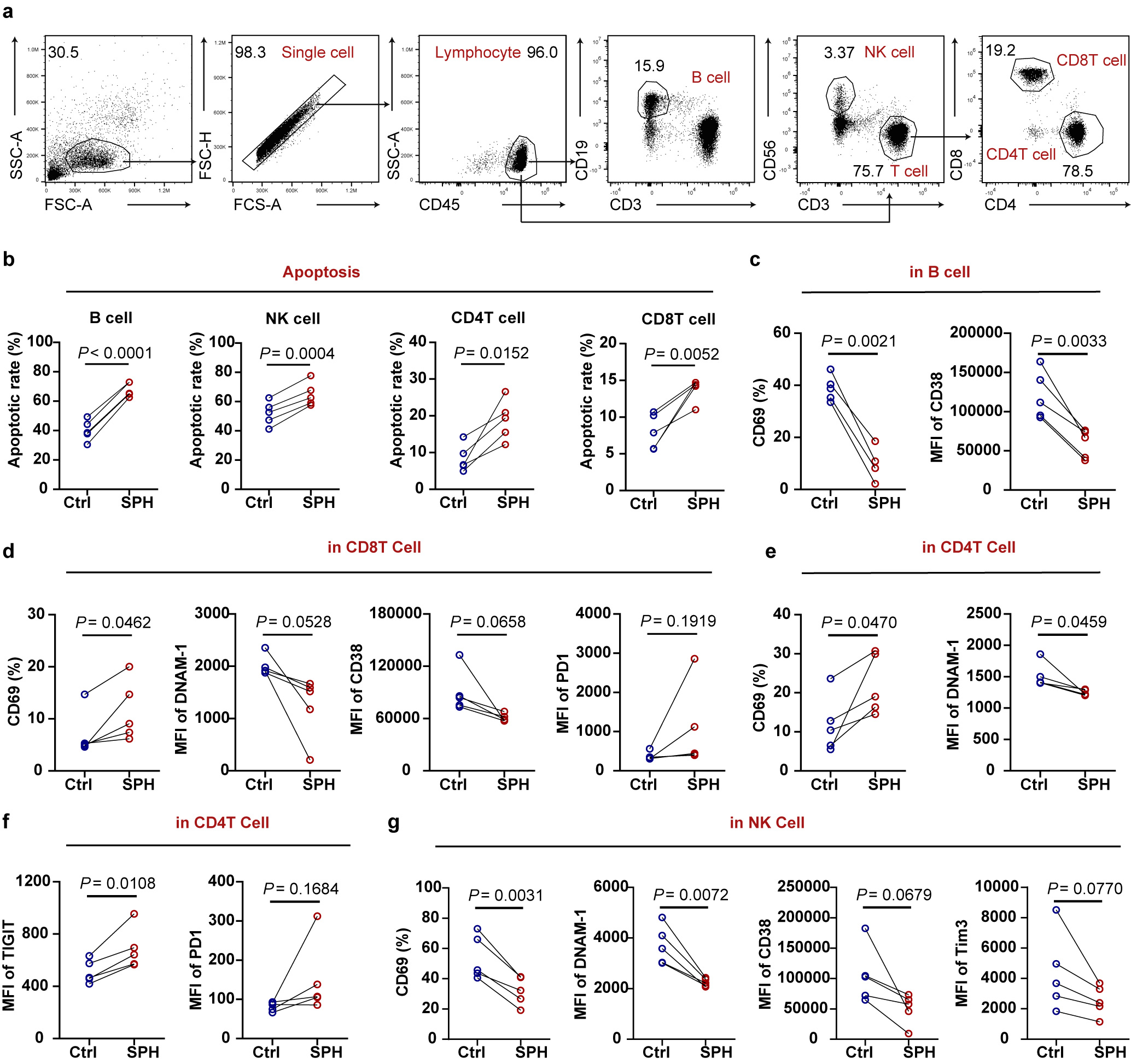


**Figure. S3. Functional analysis of cord blood mononuclear cells (CBMCs) following SPH treatment (10 μM, 24 h), related to Figure 2. a** Representative flow cytometry gating strategy for lymphocyte population, including CD4^+^T, CD8^+^T, NK and B cells. **b** Flow cytometry analysis showing the proportion of apoptotic cells across the immune populations (n = 5, biological replicates). Apoptosis was assessed by Annexin V/PI staining. **c-g** Flow cytometry analysis showing the functional molecules expression on B (**c**), CD8^+^T (**d**), CD4^+^T (**e**, **f**), and NK (**g**) cells (n = 5, biological replicates). Data were analyzed using two-tailed paired Student’s t test.

**
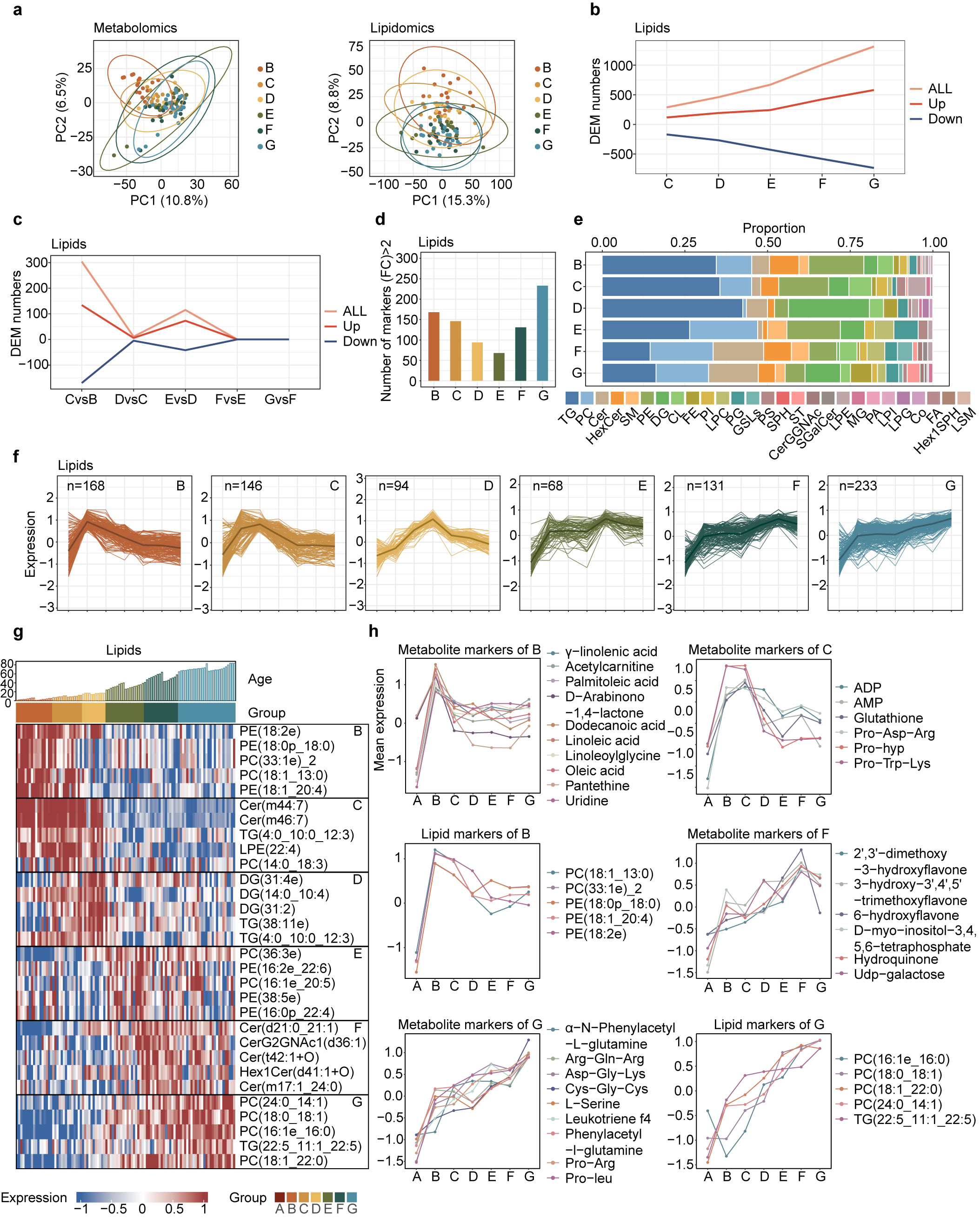
**

**Figure. S4. Significant perturbation in the plasma metabolome of newborns, related to Figure 3. a** PCA score plots for metabolomics and lipidomics in different age groups excluding group A (n=103 individuals). Each sample is colored by group information. **b** Line plot displaying the number of differentially expressed lipids for pairwise analysis in age groups, referenced to early childhood (Group B). Each line is colored by the expression trends. **c** Line plot displaying the number of differentially expressed lipids in the pairwise analysis in adjacent groups. Each line is colored by the expression trends. **d** Bar graph showing the number of differential lipids for each group compared to all other groups. **e** Scaled mean expression trajectory of marker lipids in different age groups, with markers derived from Supplementary Fig. 3d. **f** Stacked bars illustrating the different proportions of lipid classes of marker lipids in age groups. **g** Heatmap showing the scaled mean expression of selected marker lipids in age groups. Color bars indicate the age groups, the top bar plot represents age. **h** Line plot displaying scaled mean expression trajectory of marker lipids in age groups.**
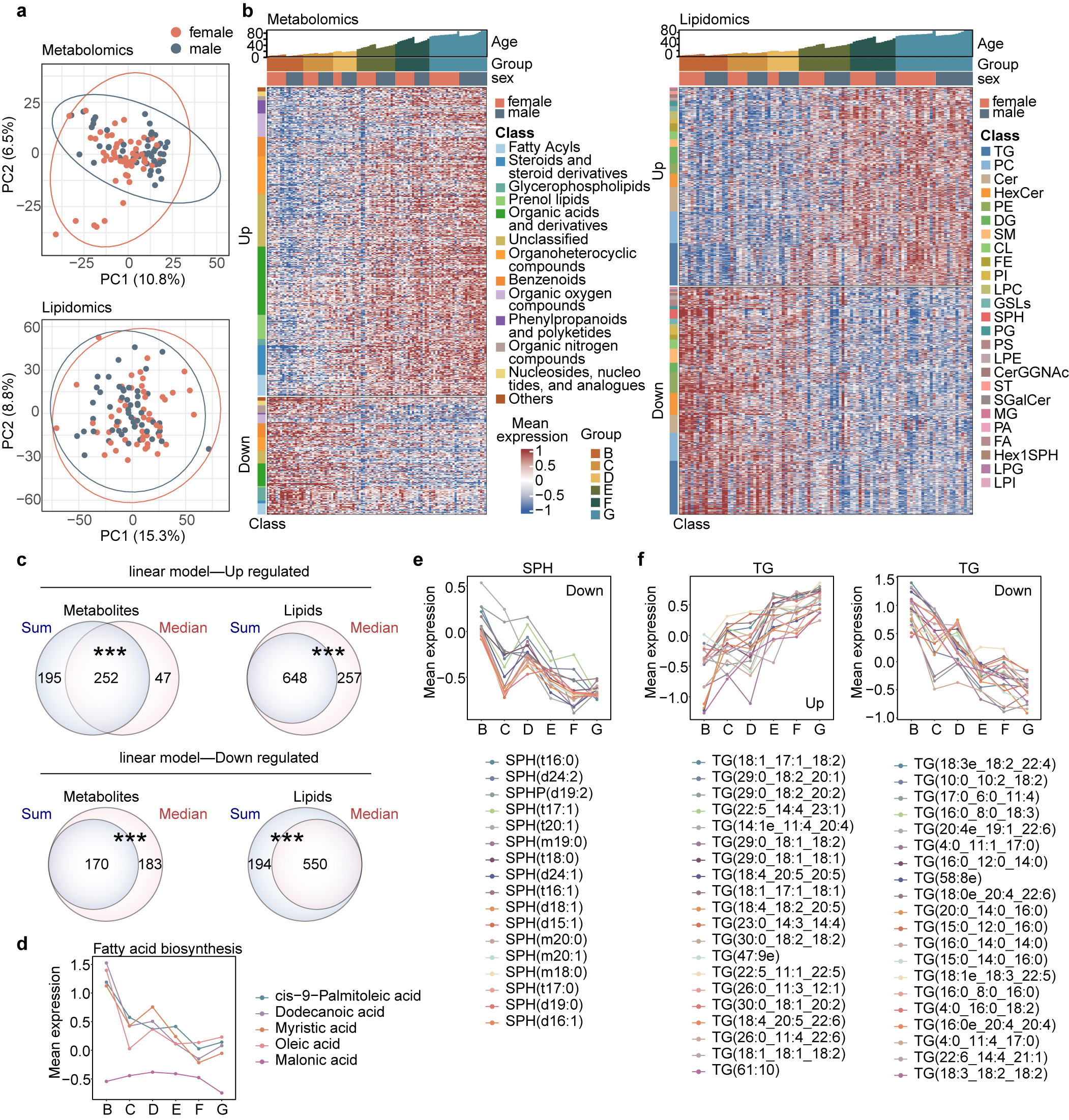
** **Figure. S5. Linear metabolomic and lipidomic changes during lifespan, related to Figure 4. a** PCA score plots for metabolomics and lipidomics on sex, including age groups, excluding group A (n=103 individuals). Each sample is colored by the sex information. **b** Scaled mean expression of enriched metabolites in the fatty acid biosynthesis pathway (fig. 4c enriched). **c** Venn diagram depicting the overlap of DEMs with linear age-related changes identified by total-sum and median normalization. One-sided Fisher’s exact test, ***P < 0.0001. **d** Heatmap of the expression of age-related metabolites (left) and lipids (right). Color bars on the left indicate the class, and the top three bar charts represent age, group, and gender, respectively. **e, f** Scaled mean expression of the top-20 lipids in SPH(d) and TG(e).**
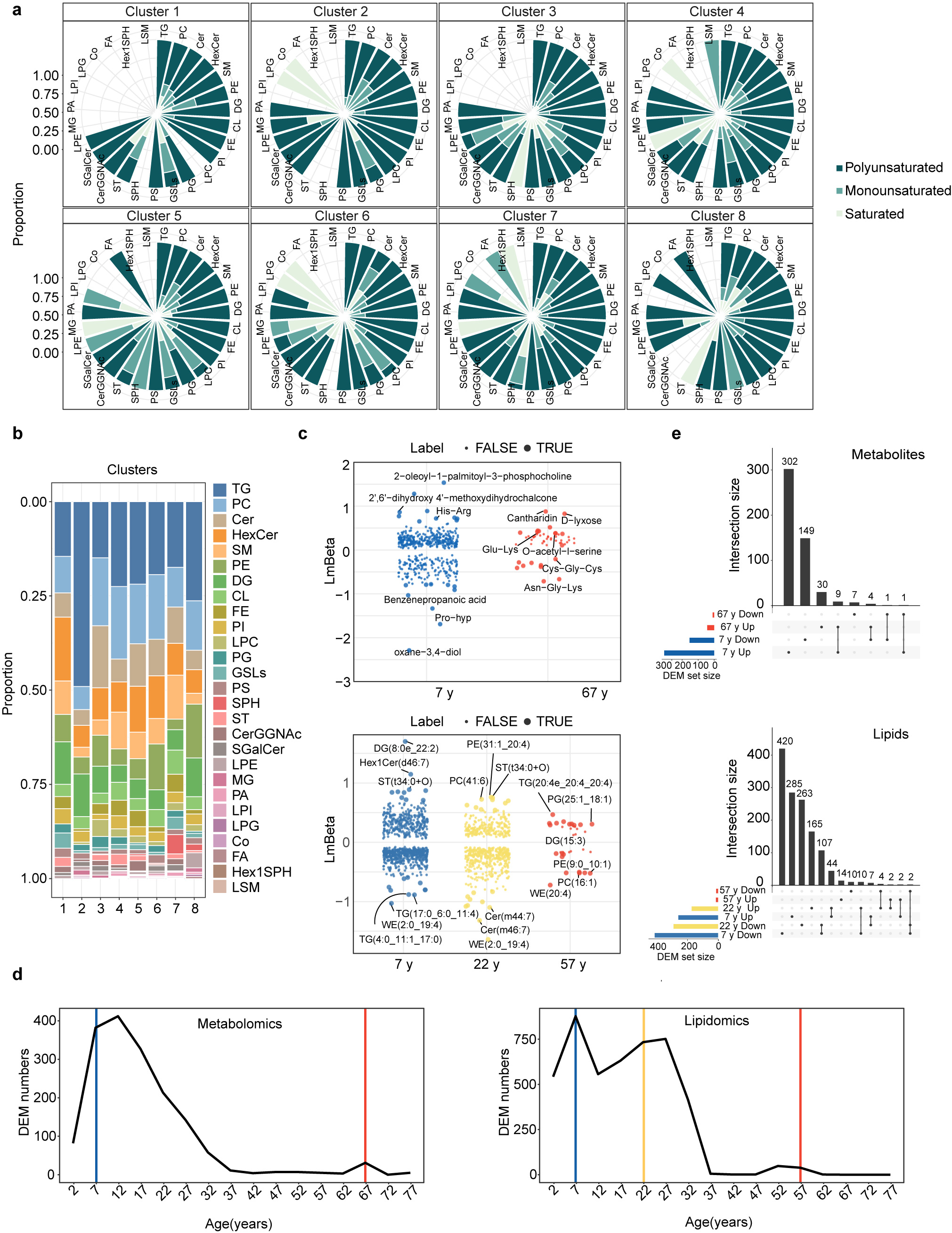
**

**Figure. S6. Nonlinear features changing with lifespan, related to Figure 5**. **a** Nightingale rose graph depicting the distribution of the total concentrations of polyunsaturated, monounsaturated, and saturated lipid types in each cluster. **b** Stacked bars illustrating the proportion of lipid classes in different lipid clusters. **c** DE-SWAN trajectories generated using median normalization, with peaks identified in total-sum normalization highlighted for comparison. **d** Manhattan plot displaying the altered metabolites identified by DE-SWAN at age 7 and 67 years (upper), and lipids at age 7, 22, and 57 years (lower). **e** UpSet plot showing the intersections between several aging peaks in metabolite (upper) and lipids (lower). Significance was tested using the F-test, followed by Benjamini–Hochberg (BH) correction.

**
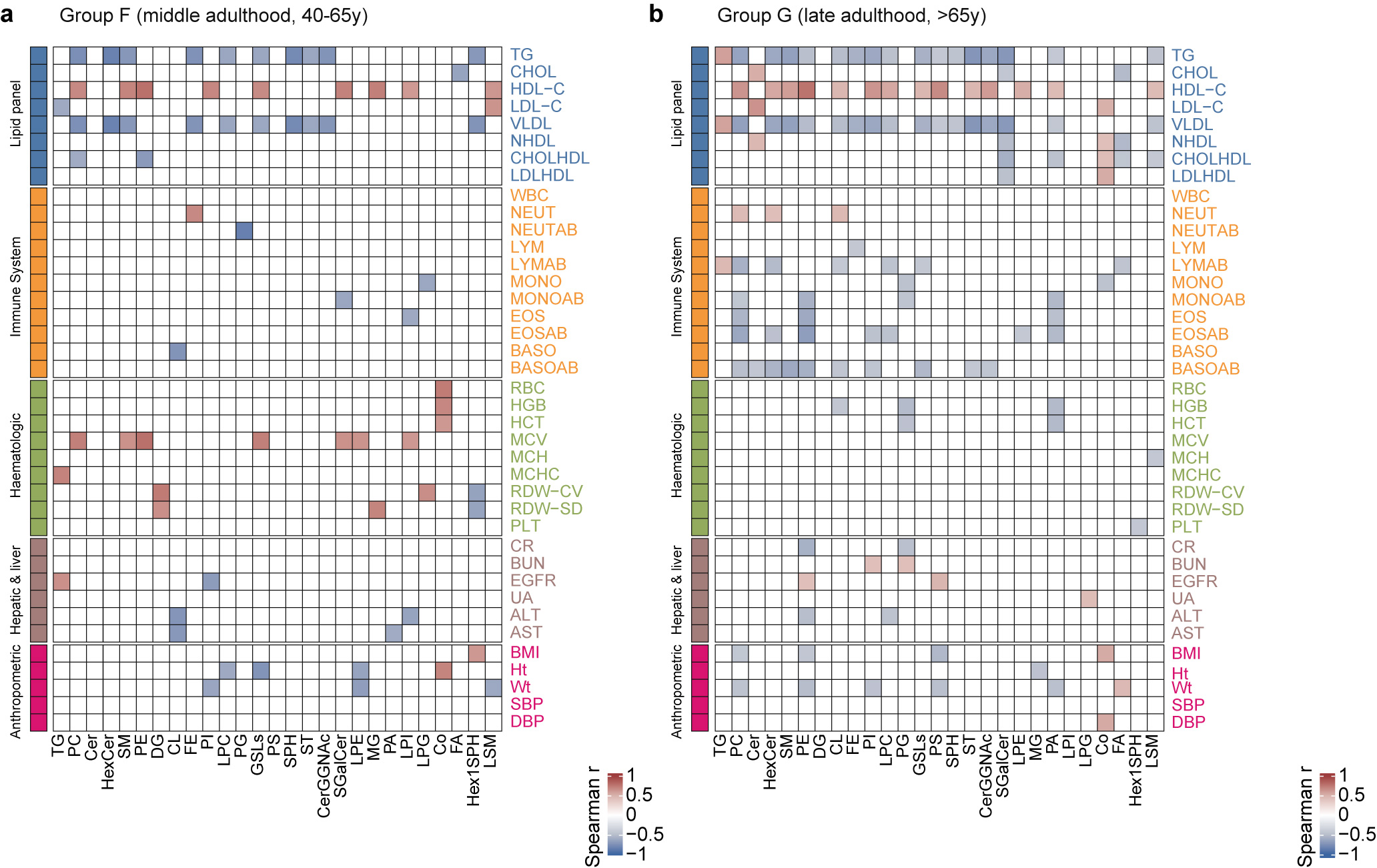
**

**Figure. S7. Linking of lipid profiles to clinical indicators of aging, related to Figure 6**. **a, b** Spearman correlation between clinical indicators and the mean expression of each lipid class in middle adulthood (Group F) **(a)** and late adulthood (Group G) **(b)**. Correlations are shown when >0, red and blue color represent positive and positive correlations, respectively.**
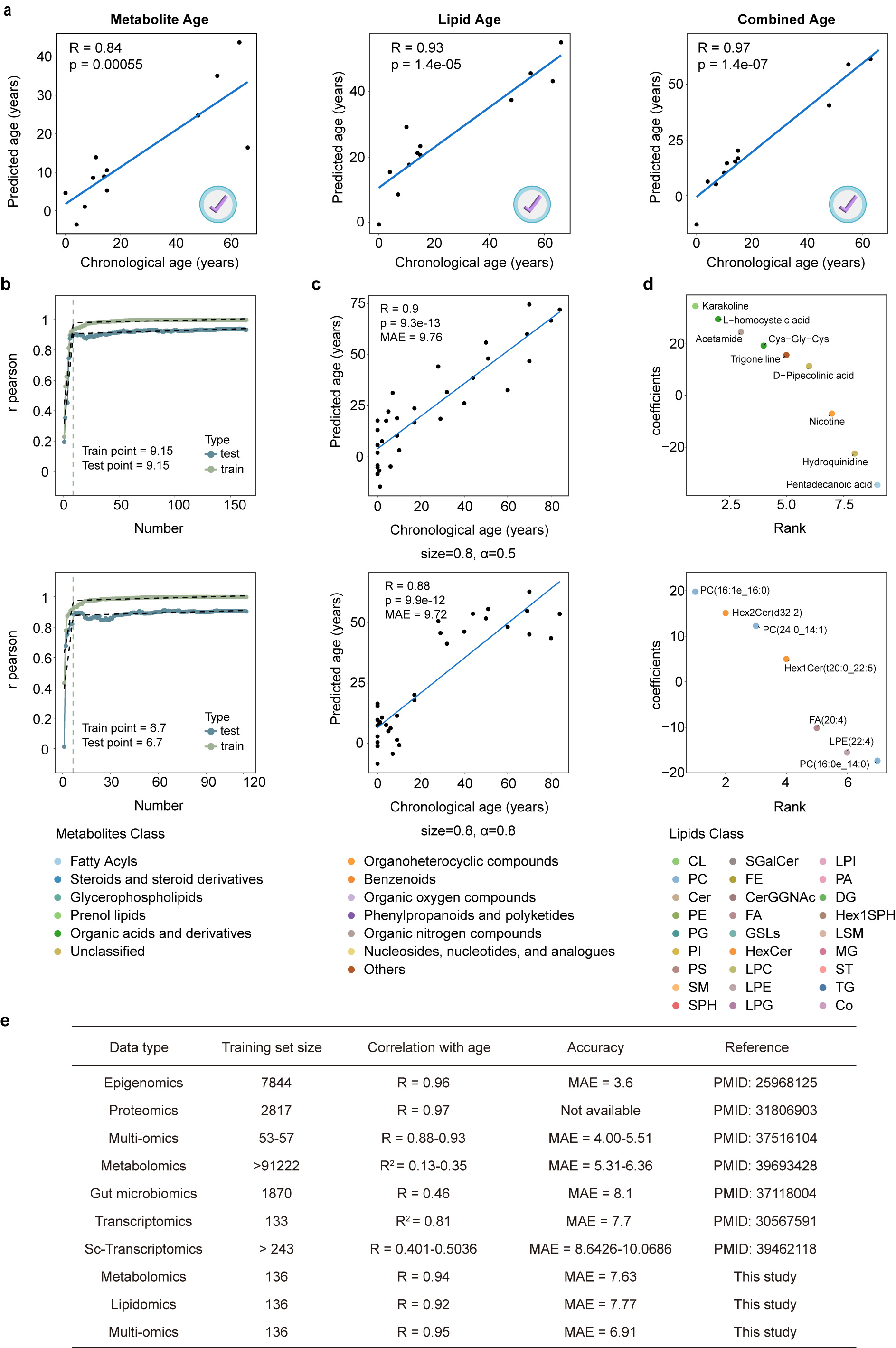
 Figure. S8. The aging clock built from metabolomic and lipidomic data, related to Figure 7**. **a** Validation of the aging clock models (metabolite, lipid and metabolite-lipid combined aging clock models) in an independent cohort (n = 12). The linear relationship between the predicted ages from the age predictors and chronological age is shown. The Pearson’s correlation coefficients (R) and p value are presented. **b** Dashed lines represent broken- stick models of metabolites (upper) and lipids (lower), the Pearson correlation coefficient (R) represents the relationship between the predicted and chronological ages. **c** The linear relationship between the predicted ages from the age predictors and chronological age are shown for the reduced metabolite model (upper) and the reduced lipid model (lower). **d** Dot plots illustrating the components of the reduced metabolite model (upper) and the reduced lipid model (lower). Dots are colored by substance classes. **e** A comparative summary of model complexity and predictive performance across previously published aging clocks.

**Table S1. Baseline characteristics of GroupA-G**

| Group | Sample | Age (year) | sex | Median age |
| --- | --- | --- | --- | --- |
| A | A1  A2  A3  A4  A5  A6  A8  A9  A10  A11  A12  A13  A14  A15  A16  A17  A18  A19  A20  A21  A22  A23  A24  A25  A26  A27  A28  A29  A30  A31  A32  A33  A34 | 0  0  0  0  0  0  0  0  0  0  0  0  0  0  0  0  0  0  0  0  0  0  0  0  0  0  0  0  0  0  0  0  0 | male  female  female  male  female  male  male  male  male  female  female  female  male  female  female  male  male  male  male  male  female  female  male  male  male  male  male  male  male  female  female  male  male | 0 |
| B | B1  B2  B3  B4  B5  B6  B7  B8  B9  B10  B11  B13  B14  B15  B16  B17  B18 | 0.83  1.083  1.17  1.83  2.083  2.75  3  3  3  3  4  4  5  5  5  6  6 | male  female  male  male  female  female  male  male  female  female  male  female  male  male  female  female  female | 3 |
| C | C2  C3  C4  C5  C6  C7  C8  C9  C10  C11  C13  C15  C16  C17 | 7  7  8  8  9  9  9  9  10  10  11  11  12  12 | female  female  male  female  male  male  female  female  male  male  female  female  male  male | 9 |
| D | D1  D2  D4  D7  D8  D9  D10  D11  D12  D13  D14 | 14  14  15  16  16  17  17  17  17  17  17 | male  male  female  male  male  female  male  male  female  female  male | 17 |
| E | E1  E2  E3  E4  E5  E6  E7  E8  E9  E10  E11  E12  E13  E14  E15  E16  E17  E18 | 24  25  26  27  28  29  30  30  31  32  33  33  35  36  37  39  39  40 | female  female  male  male  female  male  female  male  female  male  female  male  male  male  female  male  female  female | 31.5 |
| F | F1  F2  F3  F4  F5  F7  F8  F9  F10  F11  F12  F14  F15  F16  F17  F19 | 41  43  44  46  47  49  50  51  52  53  54  56  58  59  60  64 | female  male  male  male  female  male  female  male  female  male  female  female  male  female  female  female | 51.5 |
| G | G1  G2  G4  G5  G6  G7  G8  G9  G10  G11  G12  G13  G14  G15  G16  G17  G18  G19  G20  G21  G22  G23  G24  G25  G26  G27  G28 | 65  66  67  67  68  68  68  69  69  69  70  70  70  71  71  71  72  72  73  73  74  76  80  83  84  84  84 | female  male  female  male  male  female  female  male  female  male  male  female  female  female  female  male  female  female  female  male  female  male  male  female  male  male  male | 71 |

**Table S2. Clinical information used for correlation analysis**

| Clinicle | F1 | F3 | F5 | F7 | F8 | F9 | F11 | F12 | F14 | F16 | F17 |
| --- | --- | --- | --- | --- | --- | --- | --- | --- | --- | --- | --- |
| TG  CHOL  HDL-C  LDL-C  VLDL  NHDL  CHOLHDL  LDLHDL  GLU  WBC  NEUT  NEUTAB  LYM  LYMAB  MONO  MONOAB  EOS  EOSAB  BASO  BASOAB  RBC  HGB  HCT  MCV  MCH  MCHC  RDW-CV  RDW-SD  PLT  CR  BUN  EGFR  UA  ALT  AST  BMI  Ht  Wt  SBP  DBP | 1.76  5.76  1.61  3.5  0.61  4.11  3.58  2.17  5.02  7.68  37.2  2.86  56.8  4.36  4  0.31  1.7  0.13  0.3  0.02  4.83  145  43.6  90.3  30  333  12.7  42.4  126  71.3  5.08  95  388  8  23  22.77  165  62  100  67 | 3.37  6.12  1.51  3.36  1.25  4.61  4.05  2.23  8.37  5.7  47.6  2.72  45.8  2.61  5.3  0.3  0.9  0.05  0.4  0.02  4.77  150  43.3  90.8  31.4  346  13.2  43.8  280  55.7  4.89  127  348  21  23  24.54  176  76  127  76 | 0.97  6.36  1.52  4.48  0.36  4.84  4.18  2.95  5.66  7.46  60.2  4.49  33.6  2.51  5.4  0.4  0.5  0.04  0.3  0.02  4.6  144  42.3  92  31.3  340  12.9  43  312  55.1  3.48  113  260  13  17  22.81  151  52  95  57 | 0.86  3.13  1.34  1.47  0.32  1.79  2.34  1.10  5.66  4.6  60.3  2.77  32.8  1.51  5  0.23  1.5  0.07  0.4  0.02  5.14  163  46.3  90.1  31.7  352  11.9  38.6  170  69.5  3.98  112  419  22  18  26.96  169  77  128  73 | 0.91  6.16  2.03  4.24  0.34  4.58  3.03  2.09  4.94  5.64  47.8  2.7  44.7  2.52  5  0.28  1.6  0.09  0.9  0.05  4.78  151  44.6  93.3  31.6  339  12.3  42.2  247  68.9  5.42  93  328  12  21  24.84  163  66  133  83 | 1.36  5.91  1.48  3.93  0.5  4.43  3.99  2.66  7.45  5.2  58.3  3.03  31  1.61  7.1  0.37  2.3  0.12  1.3  0.07  5.63  160  48.1  85.4  28.4  333  11.8  35.7  299  87.2  6.62  93  334  37  27  25.51  168  72  125  80 | 1.41  4.77  1.03  3.22  0.52  3.74  4.63  3.13  4.79  4.8  50.8  2.44  39.2  1.88  7.7  0.37  1.7  0.08  0.6  0.03  4.49  148  40.1  89.3  33  369  12.1  38.7  275  70.5  4.62  108  398  25  27  25.35  172  75  141  77 | 1.98  6.37  1.95  3.69  0.73  4.42  3.27  1.89  5.21  4.47  45  2.01  48.1  2.15  4.7  0.21  1.8  0.08  0.4  0.02  4.47  142  41.3  92.4  31.8  344  12.5  42.1  192  68.3  5.63  92  369  28  25  24.03  154  57  128  65 | 0.96  5.74  1.97  3.41  0.36  3.77  2.91  1.73  4.94  4.48  52.7  2.36  41.3  1.85  4.7  0.21  0.9  0.04  0.4  0.02  4.44  138  41.1  92.6  31.1  336  12.9  43.4  137  55.7  4.39  105  232  16  26  21.64  152  50  157  70 | 1.6  6.87  1.74  4.54  0.59  5.13  3.95  2.61  4.18  4.57  72.6  3.32  21.7  0.99  4.4  0.2  0.9  0.04  0.4  0.02  4.4  137  40.2  91.4  31.1  341  13.7  45.8  262  68.9  4.2  88  375  27  36  25.08  161  65  130  84 | 2  4.49  1.22  2.53  0.74  3.27  3.68  2.07  5.29  6.64  59.9  3.98  33.3  2.21  4.2  0.28  2  0.13  0.6  0.04  4.43  125  38.7  87.4  28.2  323  13  41.2  121  49.5  4.86  107  323  43  35  26.04  158  65  148  60 |

| Clinicle | G2 | G4 | G5 | G6 | G7 | G8 | G9 | G10 | G11 | G12 | G13 |
| --- | --- | --- | --- | --- | --- | --- | --- | --- | --- | --- | --- |
| TG  CHOL  HDL-C  LDL-C  VLDL  NHDL  CHOLHDL  LDLHDL  GLU  WBC  NEUT  NEUTAB  LYM  LYMAB  MONO  MONOAB  EOS  EOSAB  BASO  BASOAB  RBC  HGB  HCT  MCV  MCH  MCHC  RDW-CV  RDW-SD  PLT  CR  BUN  EGFR  UA  ALT  AST  BMI  Ht  Wt  SBP  DBP | 1.16  4.37  1.17  2.77  0.43  3.2  3.74  2.37  5.17  4.59  48.1  2.21  33.6  1.54  4.6  0.21  12.4  0.57  1.3  0.06  4.65  152  42.4  91.2  32.7  358  13  43.1  156  75.6  5.34  96  327  18  24  26.57  165  75  158  91 | 0.72  4.88  1.97  2.64  0.27  2.91  2.48  1.34  5.6  4.72  64.5  3.04  29.4  1.39  4.9  0.23  0.4  0.02  0.8  0.04  4.32  137  41.4  95.8  31.7  331  12  41.9  156  48.9  6.58  102  192  17  21  21.3  165  58  172  95 | 1.5  4  1.23  2.22  0.56  2.78  3.25  1.80  5.72  6.09  64.2  3.91  25.4  1.53  8.9  0.54  1.1  0.07  0.7  0.04  4.93  153  43.7  88.6  31  350  12.2  39.6  246  98.8  7.24  72  414  28  21  29.4  174  89  133  83 | 1.55  5.34  1.22  3.55  0.57  4.12  4.38  2.91  5.03  4.58  62.6  2.87  27.1  1.24  6.1  0.28  3.5  0.16  0.7  0.03  4.42  150  43.5  98.4  33.9  345  12.4  45  192  69.8  5.76  98  373  16  23  27.97  167  78  157  78 | 1.13  5.66  1.81  3.43  0.42  3.85  3.13  1.90  4.81  3.42  45.2  1.55  48  1.64  5.3  0.18  0.9  0.03  0.6  0.02  4.44  134  40.2  90.5  30.2  333  12.1  40.2  150  57.7  4.87  96  301  17  27  23.53  170  68  125  81 | 0.85  5.04  1.72  3.01  0.31  3.32  2.93  1.75  5.67  4.83  57  2.75  38.3  1.85  3.9  0.19  0.4  0.02  0.4  0.02  4.25  131  37.8  88.9  30.8  347  12.6  41.2  264  61.3  6.58  94  299  17  28  20.54  143  42  127  78 | 5.19  6.8  1.5  3.38  1.92  5.3  4.53  2.25  5.51  6.62  55.9  3.7  34  2.25  6.2  0.41  2.7  0.18  1.2  0.08  4.1  141  41.2  100.5  34.4  342  14.7  55.3  250  62.7  2.92  101  357  68  103  21.01  169  60  126  74 | 1.44  3.26  1.43  1.3  0.53  1.83  2.28  0.91  7.61  9.07  52.9  4.79  42.4  3.85  3.1  0.28  1.3  0.12  0.3  0.03  4.38  121  36.8  84  27  329  13.1  39.6  225  53.7  4.18  97  219  29  29  26.9  159  68  160  77 | 1.54  5.77  1.53  3.67  0.57  4.24  3.77  2.40  5.51  7.29  55.6  4.05  32.2  2.35  4.8  0.35  6.4  0.47  1  0.07  5.09  152  46.1  90.6  29.9  330  13.6  46  270  77.6  3.7  93  295  19  26  26.49  153  62  122  77 | 1.25  6.26  1.66  4.14  0.46  4.6  3.77  2.49  5.05  5.62  62.5  3.51  29.5  1.66  6  0.34  1.6  0.09  0.4  0.02  4.54  143  41.4  91.2  31.5  345  13.4  45.1  228  76.8  5.96  92  366  15  22  24.75  151  61  150  89 | 4.6  7.26  1.59  3.97  1.7  5.67  4.57  2.50  5.49  4.32  49.2  2.12  41.4  1.79  6  0.26  2.5  0.11  0.9  0.04  4.53  141  41.5  91.6  31.1  340  12.2  40.6  204  62.5  7.39  91  235  29  35  29.28  149  65  129  74 |

| Clinicle | G14 | G15 | G16 | G17 | G18 | G19 | G20 | G21 | G26 | G27 | G28 |
| --- | --- | --- | --- | --- | --- | --- | --- | --- | --- | --- | --- |
| TG  CHOL  HDL-C  LDL-C  VLDL  NHDL  CHOLHDL  LDLHDL  GLU  WBC  NEUT  NEUTAB  LYM  LYMAB  MONO  MONOAB  EOS  EOSAB  BASO  BASOAB  RBC  HGB  HCT  MCV  MCH  MCHC  RDW-CV  RDW-SD  PLT  CR  BUN  EGFR  UA  ALT  AST  BMI  Ht  Wt  SBP  DBP | 2.37  6.61  1.48  4.25  0.88  5.13  4.47  2.87  5.6  7.52  66.9  5.04  27.7  2.08  2.8  0.21  1.9  0.14  0.7  0.05  4.94  150  44.1  89.3  30.4  340  11.9  38.1  274  73.3  6.28  75  470  11  18  26.44  158  66  177  121 | 1.17  4.13  1.95  1.75  0.43  2.18  2.12  0.90  4.67  3.79  54.6  2.07  39.1  1.48  4.7  0.18  0.8  0.03  0.8  0.03  4.17  126  38.6  92.6  30.2  326  12.7  43.2  243  50.7  8.47  98  243  18  24  20.54  148  45  130  72 | 1.12  5.74  1.62  3.71  0.41  4.12  3.54  2.29  5.49  7.01  61  4.28  34.5  2.42  3.6  0.25  0.6  0.04  0.3  0.02  4.44  141  41.4  93.2  31.8  341  12.2  41.8  198  50.4  6.83  98  348  23  28  26.99  154  64  178  123 | 0.83  5.63  1.71  3.61  0.31  3.92  3.29  2.11  5.82  5.02  57.1  2.87  29.5  1.48  8.8  0.44  5  0.2  0.6  0.03  4.58  150  44.5  97.2  32.8  337  12.1  43.7  245  75.7  4.33  92  388  15  23  22.78  179  73  137  89 | 0.83  3.71  1.15  2.25  0.31  2.56  3.23  1.96  5.62  3.98  60.5  2.41  25.4  1.01  7.3  0.29  6  0.24  0.8  0.03  3.55  111  32.6  91.8  31.3  340  13.9  47.3  183  74.4  9.58  73  511  45  36  26.67  162  70  140  65 | 1.31  6.7  1.6  4.62  0.48  5.1  4.19  2.89  6.5  4.82  61.2  2.95  31.5  1.52  5.2  0.25  1.7  0.08  0.4  0.02  4.28  127  38.8  90.7  29.7  327  12.9  42.4  167  62.7  7.04  90  249  17  23  22.97  149  51  186  78 | 1.11  5.49  1.54  3.54  0.41  3.95  3.56  2.30  5  4.64  49.4  2.29  43.1  2  6  0.28  1.1  0.05  0.4  0.02  3.61  122  36.1  100  33.8  388  13.2  49.1  231  55.4  5.78  94  313  34  32  20.7  163  55  121  67 | 1.72  3.21  1.01  1.56  0.64  2.2  3.18  1.54  5.55  8.43  51.6  4.35  36.2  3.05  6.4  0.54  5.1  0.43  0.7  0.06  4.45  138  41.5  93.3  31  333  12.2  41.7  219  100.6  6.31  67  300  25  22  26.61  169  76  145  74 | 1.23  4.57  1.62  2.49  0.46  2.95  2.82  1.54  5.26  4.94  71.3  3.52  19.2  0.95  6.7  0.33  1.8  0.09  1  0.05  5.16  144  43.6  84.5  27.9  330  13.1  40.2  155  80.2  4.73  82  368  12  23  19.23  166  53  156  80 | 1.55  5.4  1.32  3.51  0.57  4.08  4.09  2.66  6.16  6.48  60.9  3.95  32.1  2.08  5.6  0.36  0.8  0.05  0.6  0.04  4.58  150  42.9  93.7  32.8  350  12.4  42.9  195  75.9  5.09  84  309  17  23  23.88  165  65  189  96 | 0.92  2.92  1.27  1.31  0.34  1.65  2.30  1.03  4.4  4.96  47.8  2.37  40.9  2.03  7.3  0.36  3.4  0.17  0.6  0.03  5.23  163  48.7  93.1  31.2  335  12.6  42.8  148  91.9  5.63  69  442  25  23  24.24  165  66  161  59 |
